# On-Skin Wearable Health Monitoring Devices: Recent Trends and Perspectives

**DOI:** 10.3390/s26185770

**Published:** 2026-09-11

**Authors:** Francisco J. Romero, Isabel Blasco-Pascual, Alfonso Salinas-Castillo, Noel Rodríguez, Diego P. Morales

**Affiliations:** 1 Department of Electronics and Computer Technology, Faculty of Sciences, University of Granada, 18071 Granada, Spain; noel@ugr.es (N.R.); diegopm@ugr.es (D.P.M.); 2 Department of Analytical Chemistry, Faculty of Sciences, University of Granada, 18071 Granada, Spain; isablasco@ugr.es (I.B.-P.); alfonsos@ugr.es (A.S.-C.)

**Keywords:** e-health, energy harvesting, non-invasive sensing, sustainable electronics, wearables, wireless embedded systems

## Abstract

On-skin non-invasive Wearable Health-Monitoring Devices (WHMDs) have rapidly evolved from laboratory prototypes into commercially viable systems capable of continuously tracking physiological and biochemical signals. By integrating epidermal temperature sensors, electrophysiological electrodes, biochemical sensing platforms, low-power electronics, and wireless communication technologies, these systems are emerging as key enablers of personalized and decentralized healthcare through the continuous acquisition of clinically relevant information directly from the skin surface. In this Perspective, we present our view on the state-of-the-art across the key technological pillars that define modern on-skin WHMDs, including non-invasive sensing strategies, advanced materials, processing and wireless communication units, energy-storage solutions, energy-harvesting techniques, and power-management architectures, with a particular focus on technologies that have already reached high Technology Readiness Levels (TRLs). We highlight how the next-generation of on-skin WHMDs must balance performance with sustainability and long-term reliability. This includes the adoption of biodegradable and recyclable materials, low-power and reconfigurable electronics, solid-state batteries, and hybrid energy-harvesting systems. By aligning technological innovation with human-centric and eco-friendly design principles, on-skin WHMDs can evolve into scalable, equitable, and environmentally responsible tools for future digital healthcare.

## 1. Introduction

In recent years, advances in nanotechnology, materials science, flexible electronics, advanced sensing systems, wireless communication, and low-power circuit design have boosted a profound transformation in Wearable Health Monitoring Devices (WHMDs). These technological developments have enabled the transition from lab-scale prototypes to industrial and commercial deployment, allowing WHMDs to evolve from simple consumer accessories into advanced platforms capable of continuously monitoring a wide range of physical and biochemical indicators [1]. WHMDs are expected to transform the healthcare model by enabling ubiquitous Point-of-Care (POC) diagnostics. By providing continuous, real-time access to clinically relevant information directly, WHMDs enable earlier detection of health deterioration, faster medical intervention, and more personalized treatment strategies. This shift toward decentralized diagnostics not only improves patient outcomes, but also alleviates the burden on healthcare systems by reducing the need for frequent in-clinic assessments and reducing overall healthcare costs [2,3].

Among the different wearable healthcare technologies currently under development, on-skin devices have emerged as one of the most promising approaches due to their ability to establish continuous, non-invasive interfaces with the human body while maintaining user comfort and ease of deployment. In this context, this Perspective focuses on on-skin WHMDs that acquire physiological and biochemical information through non-invasive skin-interfaced sensing technologies. While implantable, minimally invasive, and subcutaneous wearable systems have achieved remarkable clinical performance and continue to represent an important research direction within wearable healthcare, parallel efforts are increasingly exploring non-invasive alternatives that can simplify device deployment, facilitate reusability, and improve user acceptance [4]. Non-invasive on-skin sensing, however, is not limited to electronic transduction since stimuli-responsive optical approaches, such as colorimetric hydrogel patches based on thermoresponsive polymers and mechanochromic materials [5,6], which allow for smartphone-based image readout without requiring analog front-ends or power sources, also represent a promising complementary line of research.

Within the broad scope of WHMDs, this Perspective focuses specifically on the electronic and wireless side, covering the main technologies that enable continuous, quantitative, and remotely accessible monitoring. In this context, the rapid evolution of WHMDs has been accompanied by a progressive increase in the Technology Readiness Levels (TRLs) of many of their Key Enabling Technologies (KETs), including sensing modules, wireless communication systems, antennas, power-management units, and energy-storage solutions. While many emerging concepts remain at early stages of development, several technologies discussed in this Perspective have already progressed well beyond laboratory proof-of-concept demonstrations and have reached TRLs in the 6–7 range, demonstrating manufacturability, reproducibility, and suitability for validation in relevant environments. However, the transition from individually validated components to fully integrated wearable systems capable of long-term operation under diverse real-world conditions remains an important challenge. Factors such as environmental exposure, temperature fluctuations, humidity, repetitive mechanical deformation, accidental impacts, and user-dependent variability can significantly affect device performance and reliability. Consequently, while several enabling technologies have achieved high levels of technological maturity, further efforts are still required before complete WHMD platforms can consistently reach the highest technology readiness levels (TRL 8–9) associated with large-scale deployment and routine use. As a result, current research and development efforts are particularly focused on improving the stability and sensitivity of non-invasive sensors, further miniaturizing system components, enhancing power autonomy, and enabling efficient data processing.

**Figure 1:**
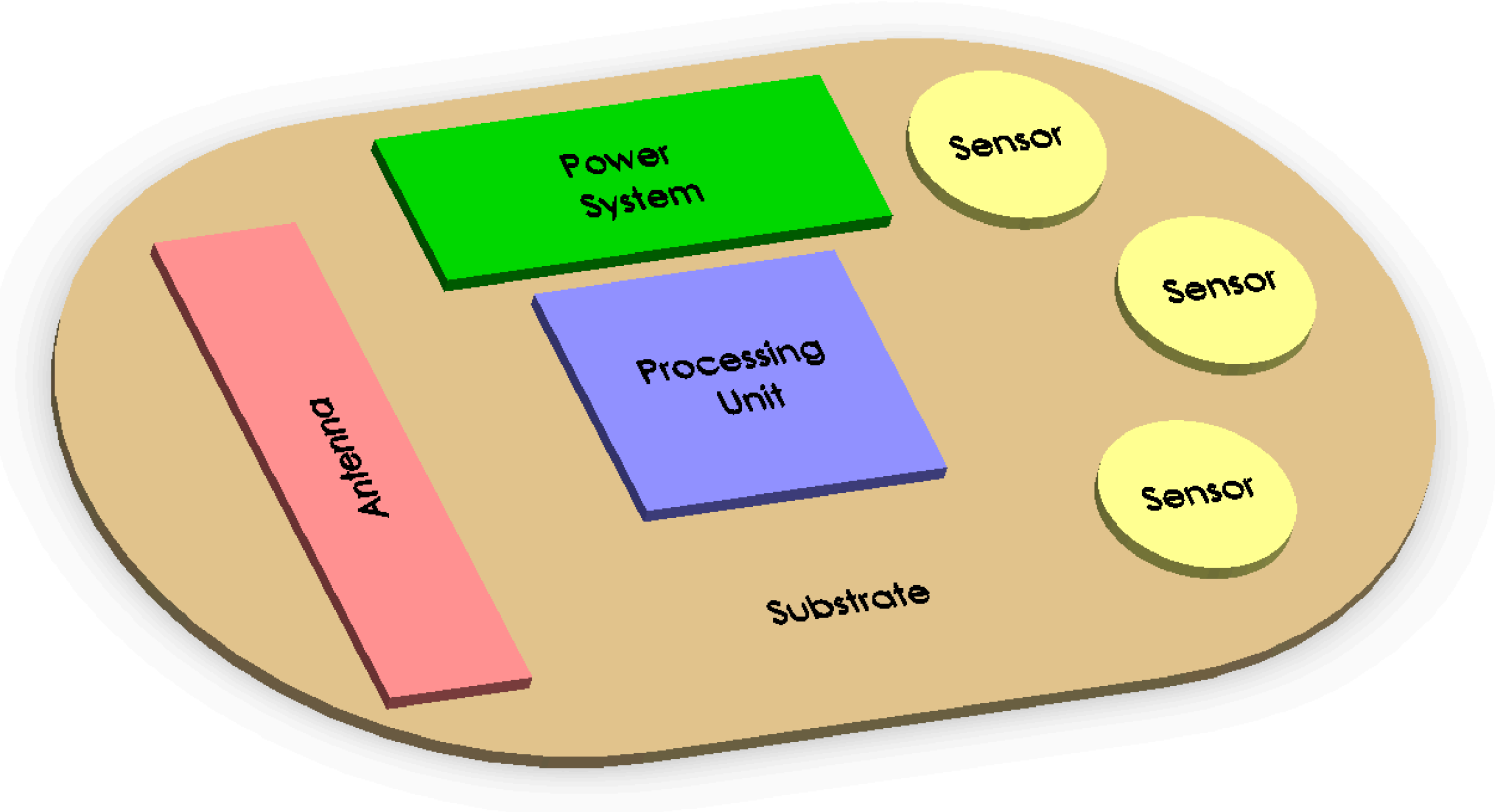
Main building blocks of a wireless WHMD including sensors, power system, processing unit(s) and antenna(s).

The manuscript is organized according to the main building blocks of a generic wireless on-skin WHMD, as illustrated in Figure 1. We consider as building blocks the fundamental hardware and technological components that typically compose an on-skin wireless WHMD, including sensing units, processing and communication units, antennas, and power systems. Rather than organizing the discussion around specific clinical applications, monitored biomarkers, or complete end-to-end systems, we adopt a technology-centered perspective focused on these core enabling elements. This approach allows us to revisit representative technologies while discussing the key engineering challenges, opportunities, and future directions associated with each technological domain. In this sense, Section 2 reviews recent advances in sensing units, covering the main biosignals and biomarkers that can currently be monitored non-invasively, as well as emerging sensor design strategies. Section 3 summarizes trends in processing units responsible for managing sensor data and enabling wireless communication. Section 4 examines how antennas are seamlessly integrated into on-skin WHMDs to support reliable data transmission. Section 5 discusses current and future strategies for power management, with particular emphasis on energy-harvesting (EH) techniques aimed at reducing battery dependency. Finally, Section 6 presents the conclusions and summarizes future perspectives for on-skin WHMD development.

## 2. Sensors

Recent advancements in on-skin WHMDs have greatly enhanced the remote and continuous monitoring of patients with chronic diseases, particularly those related to the cardiovascular system, neurological disorders, and diabetes. These capabilities are enabled by wearable sensors capable of monitoring physiological parameters such as body temperature, electrophysiological signals (e.g., electrocardiogram, electromyogram and electroencephalogram) and biochemical biomarkers, which support continuous monitoring applications, ranging from cardiovascular and metabolic disease management to neurological conditions monitoring such as epilepsy, Parkinson’s disease, sleep disorders, and neurodegenerative diseases. By providing continuous access to these physiological and biochemical indicators, WHMDs are enabling a new generation of POC diagnostic solutions that support timely medical intervention, personalized treatments, and more effective disease management [7]. However, the performance of on-skin sensors depends not only on the sensing mechanism itself, but also on the ability to establish stable, comfortable, and long-lasting interfaces with the skin. Consequently, factors such as conformability, adhesion, breathability, biocompatibility, and mechanical robustness have become increasingly important considerations in modern wearable sensing systems. In this sense, current research on sensors for on-skin WHMDs is centered on exploring alternative materials, innovative sensing mechanisms, and scalable manufacturing processes that preserve accuracy and reliability while reducing production costs. These efforts aim to facilitate broader accessibility and promote a more pervasive, patient-centered healthcare ecosystem [8]. In this context, this section presents recent trends in non-invasive wearable sensors, with a focus on sensing techniques, material innovations, and fabrication strategies. The discussion is organized around key on-skin sensor categories, including temperature sensors, electrophysiological sensors, and sweat-based biochemical sensors.

### 2.1. Temperature Sensors

Temperature is one of the most important physiological parameters, as increased skin temperature is a preliminary indicator of disease, making continuous monitoring essential in many clinical scenarios. Accurate body temperature measurement is crucial for health monitoring, but commercial contact-based sensors are typically rigid, limiting their suitability for long-term use, especially on curved body surfaces. Non-contact infrared devices offer an alternative, but they require a clear line of sight and are sensitive to surface emissivity [9]. Moreover, it should be noted that the accuracy of on-skin wearable temperature monitoring strongly depends on sensor placement. Body regions located above superficial blood vessels, such as the wrist, temple, neck, or axillary region, tend to provide measurements that are more closely correlated with core body temperature than other skin locations, as increased capillary blood perfusion enhances the effective thermal conductivity of peripheral tissues [10].

**Figure 2:**
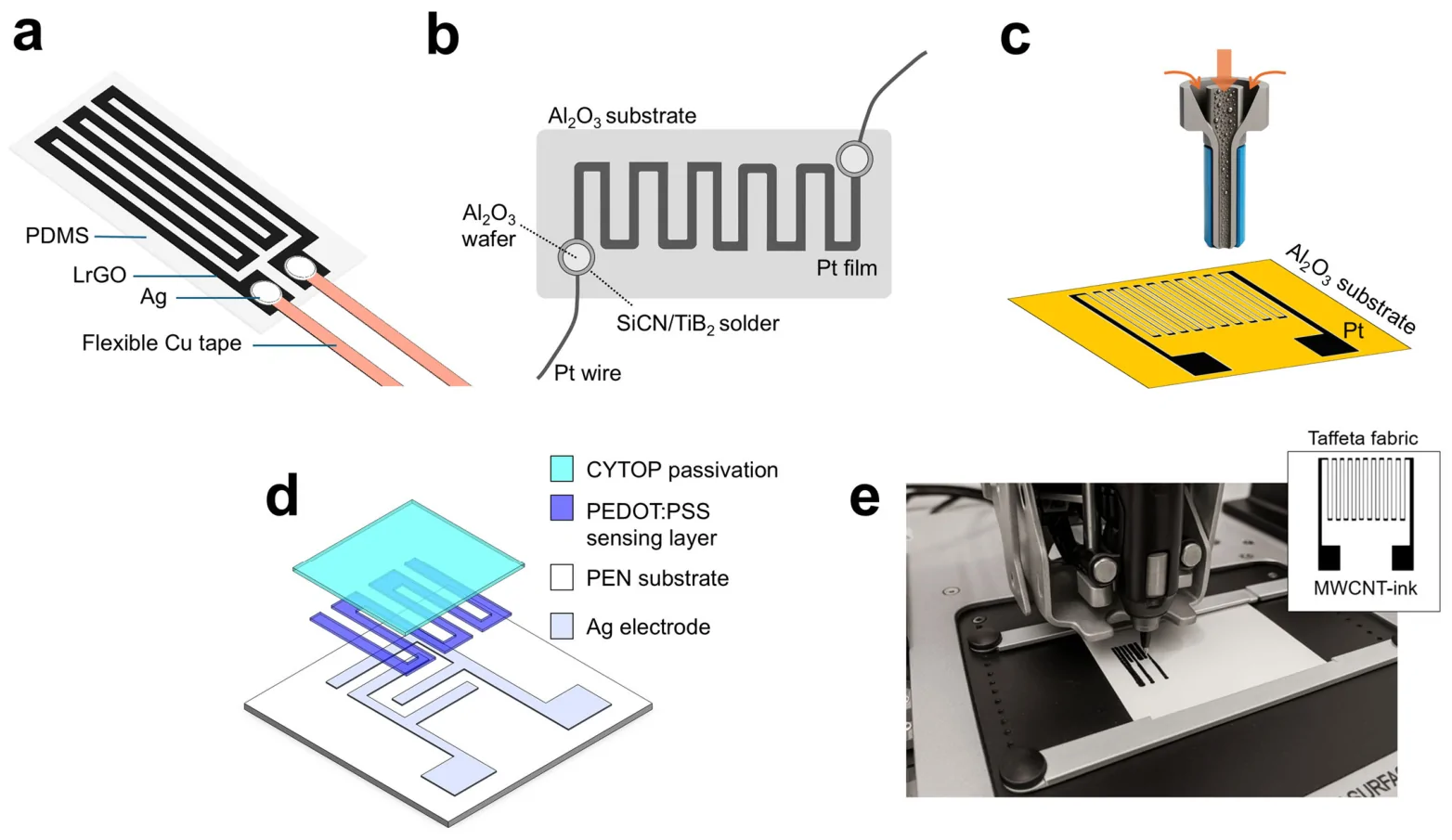
(**a**) NTC temperature sensor based on LrGO on a PET substrate and encapsulated with PDMS, adapted with permission from the corresponding author from [8]. (**b**) RTD temperature sensor based on 3D-printed Pt on an Al_2_O_3_ substrate, based on [18]. (**c**) RTD sensor based on aerosol-jet printed Ag nanoparticles on a Kapton^®^ PI substrate, based on [19,25]. (**d**) NTC sensor using an IDE structure of inkjet-printed Ag nanoparticles on a PEN substrate, with a PEDOT:PSS sensing layer and polymer passivation, based on [20]. (**e**) NTC temperature sensor based on ink-jet printed multi-walled CNTs (MWCNTs) on a textile substrate to be encapsulated with PU afterwards, adapted from [26] based on [24].

For on-skin wearable applications, the simplest temperature sensors are resistive-based devices consisting of two terminals fabricated from materials whose conductivity strongly depends on temperature (as shown in Figure 2). These sensors are generally classified into two groups: Resistance Temperature Detectors (RTDs) and Negative Temperature Coefficient (NTC) thermistors. RTDs are typically made from metals such as platinum (Pt), whose resistance increases with temperature, whereas NTC thermistors are based on metal oxides that exhibit the opposite behavior. Although Positive Temperature Coefficient (PTC) thermistors also exist, their sharp resistance increase above a specific temperature threshold makes them less suitable for WHMD applications.

In the context of commercial temperature-sensing solutions suitable for WHMDs, we can find solutions that range from NTC thermistors (e.g., the NTC-MF52 from Canadian Thermostats & Control Devices Ltd.— Montreal, QC, Canada) and analog sensors to be directly connected to an Analog-to-Digital Converter (ADC), such as the TMP3x series from Analog Devices Inc (Cambridge, MA, USA), to integrated circuits with digital interfaces like the MAX30205 sensor from Maxim Integrated Products Inc (San Jose, CA, USA). In contrast, research efforts are primarily focused on developing sensors based on flexible, stretchable, and biocompatible materials. These devices are typically fabricated on flexible substrates such as polyethylene terephthalate (PET) or polyimide (PI), or on stretchable substrates such as polydimethylsiloxane (PDMS) or poly(ethylene naphthalate) (PEN), onto which the temperature-sensitive material is deposited or synthesized [11,12]. While these substrates remain the benchmark for wearable electronics due to their well-established processing methods and mechanical reliability, increasing attention is being devoted to more sustainable and environmentally friendly alternatives. In particular, cellulose-based substrates, including nanocellulose papers and cellulose nanofibril films, have emerged as promising candidates owing to their low cost, biodegradability, breathability, and favorable mechanical properties [13]. Similarly, carbohydrate-based materials, such as chitosan or silk fibroin, are being explored as biodegradable platforms for next-generation wearable sensors [14]. Moreover, advanced polyurethane (PU)-based composites are also attracting considerable interest because their mechanical properties can be tuned across a wide range of stiffness and elasticity values while maintaining good biocompatibility and durability [15]. These emerging substrates offer opportunities to simultaneously improve conformability, user comfort, and environmental sustainability, making them promising candidates for future on-skin wearable devices.

When it comes to fabrication, two main fabrication approaches are commonly employed. When the sensing material can be patterned arbitrarily (compatible with techniques such as laser scribing or printing) it is often deposited in a serpentine geometry to enhance sensing area and flexibility. Examples include laser-processed materials such as laser-reduced graphene oxide (LrGO) and laser-induced graphene (LIG) NTC thermistors [16,17], as well as printed metal-based RTDs using Pt or silver (Ag) inks [18,19]. The second approach involves printing a metal-based interdigitated electrode (IDE) structure and subsequently depositing the temperature-sensitive material on top. This method is widely used with materials such as poly(3,4-ethylenedioxythiophene):poly(styrenesulfonate) (PEDOT:PSS) and carbon nanotubes (CNTs) [20,21]. This is also the approach followed by some vendors, such as the commercial flexible NTC temperature sensors fabricated by Advanced Thermal Technologies (ATT, Dobl-Zwaring, Austria) [19]. Representative examples of sensors fabricated using these strategies are shown in Figure 2.

In addition to the sensing material and substrate, the encapsulation layer plays a critical role in wearable temperature sensors. It must provide efficient thermal conduction while protecting the device from humidity, sweat, and mechanical wear, all of which can compromise measurement accuracy and long-term stability. Common encapsulation materials include elastomers such as PDMS, PU, and Ecoflex, among others [22,23,24].

### 2.2. Electrophysiological Sensors

**Figure 3:**
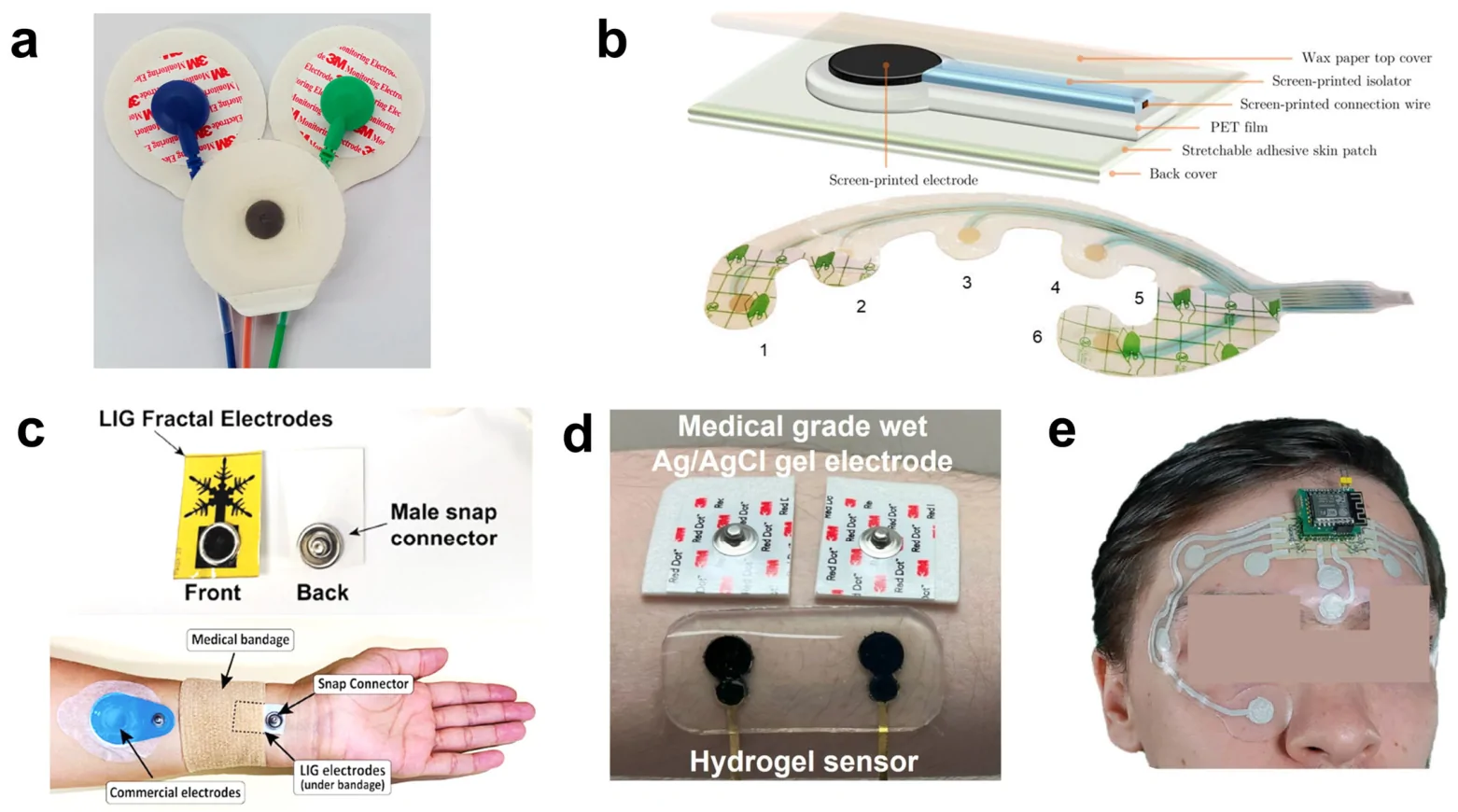
Body surface electrodes: (**a**) Commercial Ag/AgCl electrode with solid-gel electrolyte from 3M Health Care (Seoul, Republic of Korea), adapted from [37]; (**b**) carbon-based screen-printed ECG electrode and EOG patch with six (1–6) electrodes on a stretchable adhesive skin patch, adapted from [38]; (**c**) dry LIG electrode on adhesive Kapton^®^ PI film attached to a paper substrate with a standard 4 mm snap connector, adapted from [34]; (**d**) ECG and EMG electrodes based on conductive polymer polypyrrole (PPy) embedded on hydrogel (ecoflex and adhesive PDMS), adapted from [36]; (**e**) printed EEG/EOG/EMG patch based on conductive Ag–In–Ga–SIS ink over a Thermoplastic polyurethane (TPU) film, adapted from [39].

Electrophysiological sensors translate the bioelectrical activity generated by tissues and cells into measurable biopotentials. The most common biopotential recordings, including electrocardiogram (ECG), electromyogram (EMG), electroencephalogram (EEG), and electrooculogram (EOG), are fundamental tools for health monitoring, as they provide valuable physiological information in a non-invasive manner. These signals are typically acquired using biopotential electrodes placed on the skin surface. The gold standard for biopotential acquisition is based on silver/silver chloride (Ag/AgCl) electrodes. This kind of electrode consists of a self-adhesive foam substrate that integrates the Ag/AgCl sensor, typically with a round shape with 1 cm of diameter, as shown in Figure 3a. These electrodes are wet electrodes, as they rely on the use of an electrolyte (either gel or solid) to decrease the skin-electrode contact impedance and reduce motion artifacts. However, recent research has increasingly focused on dry electrodes to eliminate the need for electrolytes, thereby avoiding issues related to gel dehydration, skin irritation, and long-term stability [27]. In this direction, particularly promising are carbon-based nanomaterials such as CNTs [28,29], rGO [30,31], and LIG [32,33,34], among others. These materials, in addition to being cost-effective, have the advantage of being compatible with multiple flexible and conformable substrates, ranging from plastic substrates to textiles and foams. It is also demonstrated that these kinds of electrodes offer good skin-electrode contact thanks to a structure that allows for adapting to the skin surface. However, this also makes contact resistance more susceptible to electrode pressure [35]. Beyond common dry and wet electrodes, conductive hydrogel-based bioelectronic interfaces have also emerged as a promising intermediate approach. These materials combine high ionic conductivity with mechanical properties that more closely match those of biological tissues, enabling improved conformal contact with the skin while reducing motion artifacts and skin-electrode impedance. Given that hydrogel-based systems can retain moisture over extended periods, they also provide more stable recordings than traditional gel-based electrodes while maintaining greater comfort during long-term use [36].

In this direction, current research on biosignal electrodes for WHMD focuses on improving skin–electrode contact and mechanical properties for long-term use. Examples of body-surface electrodes for electrophysiological sensing fabricated using different materials and approaches described are shown in Figure 3.

### 2.3. Sweat Sensors

In recent years, WHMDs have also enabled the possibility of providing information at a deeper, molecular level, capabilities that were traditionally limited to invasive blood extractions. With the rapid advancement in chemical sensing technologies, the detection of analytes in accessible biofluids has opened the new opportunities for the wearable monitoring of the body’s biomolecular state. Blood remains the gold standard for clinical analyte monitoring; however, it requires invasive sampling. Recent developments in minimally invasive sensors, such as microneedle-based devices and subcutaneous implants, have demonstrated strong performance while reducing skin penetration [40,41]. A clear example includes continuous glucose monitors (CGMs), which are already commercialized by multiple companies to support diabetes and prediabetes management.

The next generation of WHMDs, however, is expected to increasingly complement invasive and minimally invasive approaches with fully non-invasive biomarker-monitoring technologies. Among the various candidate biofluids for that (such as tears, urine, and saliva), sweat stands out as the most promising for wearable applications. It is demonstrated that sweat plays a key role in thermoregulation, immune defense, electrolyte and pH balance. Moreover, it contains a rich array of chemical biomarkers, including glucose, lactate, ethanol, and cortisol (among others), which can provide valuable insights into the body’s physiological and metabolic state [42,43].

**Figure 4:**
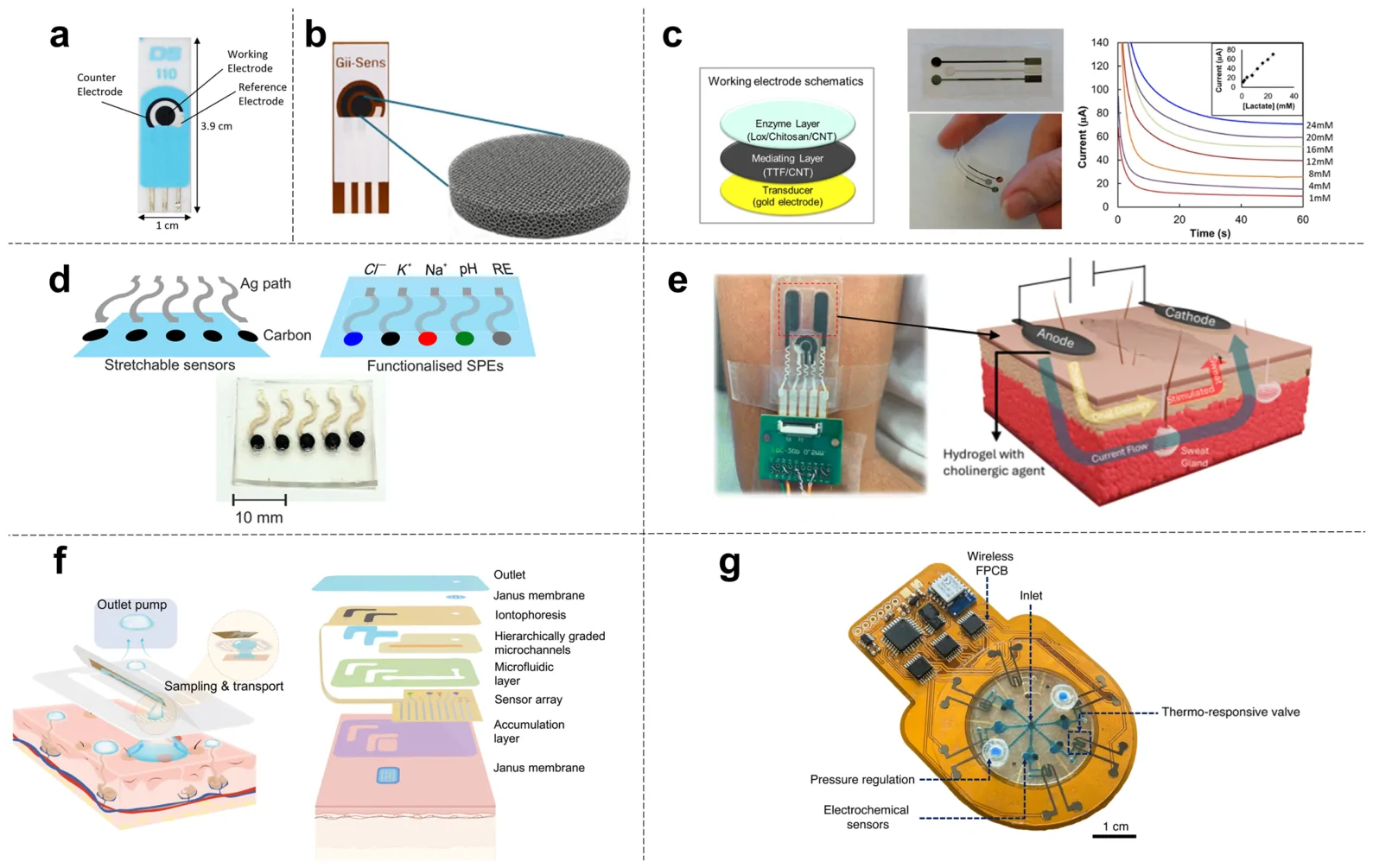
(**a**) Commercial carbon-based screen-printed electrode on a rigid ceramic substrate from Metrohm DropSens (Asturias, Spain), extracted from [48]. (**b**) Commercial electrode based on a graphene-based 3D foam on a flexible PI substrate from Gii Sens (UK), adapted from [49]. (**c**) Screen-printed carbon-based electrode on a PET substrate functionalized with a lactate-sensitive membrane optimized for the physiological range of lactate found in sweat, adapted from [50]. (**d**) Functionalized wearable electrode array for the detection of pH, Cl^−^, K^+^, and Na^+^ based on screen-printed carbon electrodes on a PU substrate, adapted from [45]. (**e**) Iontophoresis module and electrochemical cell based on LIG on a PU substrate for glucose detection, adapted from [47]. (**f**) Schematic representation of a wearable microfluidic sweat-sensing platform integrating hierarchical microchannels, iontophoresis-based sweat stimulation, and controlled sweat transport, adapted from [51]. (**g**) Integrated hydrogel-based microfluidic platform combining electrochemical sensors, fluidic routing, and thermo-responsive flow regulation for continuous sweat monitoring, adapted from [52].

Electrochemical sensors are the preferred approach for extracting analytical information from sweat, by converting electrochemical reactions into quantifiable electrical signals. As shown in Figure 4a,b, they typically consist of three electrodes: a reference electrode (RE) that maintains a stable potential, a counter electrode (CE) that completes the circuit with the solution, and a working electrode (WE) that transduces the electrochemical reaction. The WE must be functionalized with specific chemical groups or biological recognition elements, such as enzymes or antibodies, to enable selective detection of the target analyte [44]. Among the various electrochemical techniques, chronoamperometry is widely used due to its simplicity and ease of data processing [42]. In this method, a constant voltage is applied to the WE to drive the redox reaction, with the appropriate voltage typically determined via cyclic voltammetry (CV). Once the reaction begins, the resulting current between the WE and CE is monitored over time. This current generally decreases as electroactive species near the electrode surface are consumed, and both the decay profile and steady-state current can be directly correlated with analyte concentration (see Figure 4c). In addition to chronoamperometric detection, potentiometric sensors are also widely used in wearable sweat monitoring. In that case, ion-selective electrodes are used to measure the potential difference between the WE and RE according to the Nernst equation, enabling the detection of ions such as Na^+^, K^+^, Cl^−^, and pH [45]. Operating under equilibrium conditions, potentiometric sensors require minimal power and do not consume the analyte, making them well-suited for continuous electrolyte monitoring and complementary to chronoamperometric sensors targeting redox-active metabolites. An example of these sensors is shown in Figure 4d.

As in many other cases, electrochemical sensors have also benefited from advances in fabrication techniques and nanomaterials, enabling smaller devices with higher sensitivity, improved selectivity, broader dynamic range, and lower production costs. In this context, carbon-based materials are also particularly promising, not only because of their biocompatibility, but also for the ease of surface modification, functionalization and good electron transfer kinetics [46]. Therefore, as in previous sections, electrodes based on carbon-based pastes, CNTs and laser-synthesized nanomaterials over flexible and conformable substrates are extensively being investigated for the wearable monitoring of multiple species in sweat. Furthermore, in addition to advances in electrode materials and fabrication strategies, recent research has also emphasized the importance of controlled sweat stimulation to ensure reliable sampling. Among the different approaches, iontophoresis-based systems have gained particular interest. These systems use a mild electrical current to deliver cholinergic agents through the skin to activate sweat secretion in a localized and reproducible manner, as illustrated in Figure 4e. This technique, already established in clinical diagnostics, is now being adapted to wearable platforms to guarantee continuous sweat availability even under resting conditions [47]. Integrating iontophoresis modules with electrochemical sweat sensors is therefore considered a promising strategy to overcome the variability of natural sweating and enable more consistent biomarker monitoring in real-world scenarios.

Furthermore, ensuring controlled sweat generation does not by itself guarantee reliable biomarker monitoring. Once sweat is produced, it must be continuously collected, transported, and renewed at the sensing interface. Otherwise, the accumulation of previously collected sweat and reaction by-products may lead to signal drift, reduced temporal resolution, and inaccuracies in the measured biomarker concentrations [53]. Consequently, increasing attention is being devoted to the integration of microfluidic architectures within wearable sweat-sensing platforms. As illustrated in Figure 4f, recent studies have demonstrated that microfluidic networks can enable controlled sweat sampling, routing, compartmentalization, and renewal, thereby improving sampling consistency and facilitating more reliable time-resolved biochemical monitoring [51,54]. In parallel, hydrogel-based fluid-management strategies are emerging as attractive alternatives and complementary approaches due to their ability to absorb, transport, and regulate biofluid flow while maintaining intimate mechanical contact with the skin. A representative example is shown in Figure 4g [52], where hydrogel-assisted fluidic systems are used to continuously wick sweat away from the sensing region while promoting sample renewal and reducing analyte accumulation. Therefore, future wearable sweat sensors will likely evolve toward more integrated platforms in which electrochemical sensing, sweat generation, and biofluid-management functions are simultaneously optimized to enable reliable continuous monitoring.

### 2.4. Challenges and Future Directions

Despite the remarkable progress achieved in recent years, several challenges remain before this new generation of on-skin sensors can achieve the robustness and reliability for long-term deployment in real-world healthcare applications.

Beyond the sensing mechanism itself, one of the most critical aspects is the interface between the wearable device and the skin. Current flexible sensors are typically fabricated on soft and conformable substrates; however, most devices still require external tapes, adhesives, straps, dedicated mounting systems to maintain intimate contact with the skin. This additional layer introduces complexity and may reduce user comfort during prolonged use. Consequently, the development of self-adhesive epidermal sensors capable of forming stable and reversible skin interfaces represents an important research direction. Such systems should not only provide sufficient adhesion to ensure reliable signal acquisition but also preserve skin integrity during long-term operation and repeated use. Future efforts should therefore focus on multifunctional substrates and encapsulation materials that combine adhesion, flexibility, biocompatibility, and mechanical durability. In this context, conductive and bioadhesive hydrogels are emerging as particularly attractive candidates because they simultaneously provide mechanical compliance, ionic conductivity and self-healing capabilities with tissue-like interfaces, which can significantly improve long-term device integration with the skin [55]. Therefore, from a materials perspective, future on-skin sensors should progressively migrate from conventional petroleum-derived substrates toward mechanically robust bio-based composites capable of simultaneously providing flexibility, biocompatibility, breathability, and environmentally responsible end-of-life disposal.

Closely related to adhesion is the challenge of breathability. Long-term occlusion of the skin may lead to sweat accumulation, irritation, inflammation, and deterioration of the skin-device interface. While increased moisture can improve contact quality in some applications, excessive sweat accumulation may compromise measurement stability, accelerate material degradation, and reduce user comfort. For example, electrophysiological sensors may initially benefit from reduced skin-electrode impedance due to increased moisture and electrolyte concentration, but prolonged sweat exposure can also increase signal variability and motion-related artifacts. Similarly, temperature sensors are susceptible to local thermal disturbances associated with moisture accumulation and changes in evaporative cooling mechanism. Accordingly, future epidermal platforms should not only pursue mechanical conformability but also incorporate breathable architectures capable of preserving the natural physiological functions of the skin. For instance, recent developments in conductive hydrogels have demonstrated that properties traditionally considered mutually exclusive, such as stretchability, adhesion, conductivity, and environmental robustness, can be simultaneously achieved. For example, superhydrophobic conductive hydrogels capable of maintaining stable sensing performance under humid, corrosive, or contaminating environments suggest that future wearable sensors may become significantly more resilient to harsh operating conditions than current solutions [56].

For each of the specific sensors presented, important challenges remain. In the case of dry electrodes, although they have emerged as promising alternatives to conventional gel-based Ag/AgCl electrodes, they remain particularly sensitive to variations in skin contact, body motion, and mechanical deformation. Achieving low contact impedance while preserving comfort and wearability remains a delicate balance. In this context, conductive hydrogel-based interfaces also represent a particularly promising research direction, as their tissue-like mechanical properties and intrinsic ionic conductivity can improve conformal skin contact while reducing motion artifacts. However, challenges related to dehydration, long-term stability, mechanical durability, and large-scale manufacturability still need to be addressed before widespread deployment [57,58].

For sweat-based sensing, one of the main barriers to clinical adoption is associated with the complexity of the biological matrix. While sweat contains a wealth of physiological information, it is also characterized by significant variability in composition, secretion rate, pH, hydration state, and electrolyte concentration. These factors can influence sensor response independently of the target biomarker concentration. In particular, the high concentration of ions present in sweat can alter local conductivity and interfere with electrochemical measurements, particularly in amperometric and chronoamperometric sensing platforms [59]. Moreover, most of these platforms rely on enzymes, antibodies, molecularly imprinted polymers, or functional surface chemistries that gradually degrade due to biochemical reactions, environmental exposure, mechanical stress, and repeated operation. This degradation can lead to sensitivity drift, reduced selectivity, and shortened device lifetime. As a consequence, future wearable electrochemical systems should shift towards multi-analyte detection and incorporate compensation mechanisms capable of accounting for changes in sweat composition and environmental conditions. In this regard, machine-learning (ML)-based calibration and drift-compensation approaches are increasingly being explored to correct for sensor aging, cross-sensitivity, and inter-subject variability in real time, reducing the need for frequent manual recalibration. By continuously learning from multiplexed sensor arrays and reference measurements, such approaches have already been demonstrated for tracking rising and falling trends in sweat biomarkers such as cortisol and may help maintain measurement accuracy over extended wear periods even as sweat composition and secretion dynamics fluctuate [60,61].

Beyond the sensing chemistry itself, the integrated platforms envisioned in Section 2.3 combining electrochemical sensing, iontophoretic stimulation, and microfluidic sweat management introduce their own challenges. On-body iontophoresis requires careful control of current density and exposure time to reliably induce sweating without causing skin irritation or discomfort, and its performance can vary with skin hydration and individual physiology. Similarly, microfluidic and hydrogel-based sweat-handling architectures must maintain reliable, clog-free operation over multi-day wear, a requirement that has not yet been extensively validated outside controlled laboratory conditions. Addressing these reliability and safety considerations will be essential for translating integrated sweat-sensing platforms from proof-of-concept demonstrations into practical, continuously worn devices.

A further consideration that cuts across all three sensor categories discussed in this Perspective is the long-term biocompatibility and safety of composites that are increasingly employed as sensing and electrode materials. While these materials offer clear advantages in cost, processability, and electrochemical performance, questions remain regarding potential nanomaterial leaching, skin sensitization, and cytotoxicity under chronic, repeated-use conditions. Establishing standardized long-term biocompatibility testing protocols for these materials will be an important step toward their clinical and consumer adoption.

Finally, from our perspective, improvements in materials alone are unlikely to completely solve the challenges associated with on-skin sensors. Future developments will increasingly require the combination of optimized materials with sensor fusion approaches and both hardware- and software-level mitigation strategies. For instance, future on-skin temperature sensors should move beyond simple skin-temperature measurements and evolve toward intelligent systems capable of estimating core body temperature through physiological compensation models and multimodal sensor fusion. Recent studies have demonstrated the potential of combining skin temperature measurements with physiological and environmental variables such as heart rate, ambient temperature, and relative humidity through thermophysiological models to estimate core-temperature changes under real-world conditions [54]. Furthermore, multisensor fusion approaches based on skin temperature, heat flux, and heart-rate measurements combined with Kalman-filter algorithms have achieved highly accurate core-temperature estimations across different environmental and activity conditions [55]. More recently, researchers have also demonstrated that incorporating thermal-contact-resistance compensation can significantly improve estimation accuracy under practical wearable scenarios, where variations in sensor-skin contact pressure may otherwise introduce substantial errors [56]. These results highlight that future wearable temperature monitoring may depend as much on data processing, physiological modeling, and sensor fusion as on the sensing element itself. In the case of electrophysiological monitoring, shielded-drive circuit designs [62] and high-input-impedance and adaptive analog front ends [63] may help reduce motion-induced disturbances and variations in the skin-electrode interface at the hardware level. At the system level, advanced signal-processing techniques capable of identifying and mitigating motion artifacts in real time using Inertial Measurement Units (IMUs) are becoming a widespread approach [64,65]. Therefore, ML-assisted denoising, adaptive filtering, sensor-fusion strategies, and artifact-aware acquisition systems may become as important as advances in electrode materials themselves for enabling reliable long-term recordings in unconstrained environments.

Looking ahead, we believe that the next generation of on-skin sensors will be characterized by seamless skin integration, multimodal sensing capabilities, and enhanced robustness against environmental and physiological variability. Rather than pursuing maximum sensitivity as an isolated metric, future developments should prioritize reliability, long-term stability, user comfort, and clinical relevance. Achieving these objectives will require a co-design approach in which materials science, device engineering, signal processing, and clinical validation evolve together. Only through such integration will on-skin sensors successfully transition from promising laboratory demonstrations to ubiquitous healthcare technologies capable of supporting continuous and personalized monitoring in everyday life.

## 3. Processing and Communication Units

The processing unit of WHMDs is responsible for acquiring sensor data, performing the necessary signal processing, and transmitting information wirelessly to external devices. When facing the design of a WHMD, the selection of the proper processing unit (or units) involves several factors:

- *Size*: Device size is often the most significant constraint, as it directly impacts on the available space for the energy storage element (e.g., battery or supercapacitor). In some cases, the design may even be limited to battery-less implementations relying on energy-harvesting techniques.
- *Sensors output*: The processing unit must support the specific interfaces required to read sensor outputs. Most modern integrated circuits (ICs) incorporate a microcontroller unit (MCU) with analog and digital peripherals, including ADCs digital-to-analog converters (DACs), and communication interfaces such as I^2^C and SPI for connecting external analog front-end (AFE) systems. These features simplify the integration of complex sensing applications, such as ECG or electrochemical biosensors requiring signal conditioning.
- *Data transfer rate*: Different applications demand both different sampling and data transfer rates. For instance, a temperature or glucose monitoring application requires low sampling and data transfer rates, resulting in minimal power consumption. In contrast, ECG or EMG monitoring requires high sampling rates and rapid data transmission, increasing both processing load and energy consumption. It is also important to consider whether the application requires real-time processing to implement medical alerts or critical condition monitoring. Ultimately, the variable to be monitored and how the data is transmitted to external devices is strongly related to power consumption and communication protocol used by the WHMD.
- *Communication scenario*: The communication scenario defines the area that should cover the communication of the WHMD, or the devices the WHMD must be compatible with. If data is transmitted to a nearby smartphone or hub in a continuous way, Bluetooth Low Energy (BLE) can be considered. If only eventual non-periodic measurements are required, Near Field Communication (NFC) could be enough. Alternatively, Low Power Wide Area Network (LPWAN) using the standard cellular network may be required in cases of remote monitoring applications.

In the context of communication protocols, NFC and Radio Frequency Identification (RFID) based implementations are gaining increasing attention for those WHMDs that require eventual measurements. In these systems, measurements are triggered when a reader is brought near the device, coupling with the antenna. The major advantage of this approach is that the reader can power the device while establishing communication, enabling fully battery-less operation. There are multiple vendors that offer different alternatives based on this approach. For instance, the NTAG I2C family of NXP Semiconductors (Eindhoven, The Netherlands) and the ST25 dynamic NFC tags from ST microelectronics (Ginebra, Switzerland) combine a passive NFC interface with a master I^2^C to interface directly I^2^C-compatible sensors. There are other chips that integrate additional peripherals, such as SPI and ADCs, thus expanding compatibility with a wider range of sensors, like the RF430FRL152H NFC Sensor Transponder from Texas Instruments (Dallas, TX, USA). More recently, more powerful NFC-compatible ICs have emerged, offering the versatility of a microcontroller combined with NFC connectivity and energy-harvesting capabilities. An example is the NGC1081 device from Infineon Technologies AG (Munich, Germany).

**Figure 5:**
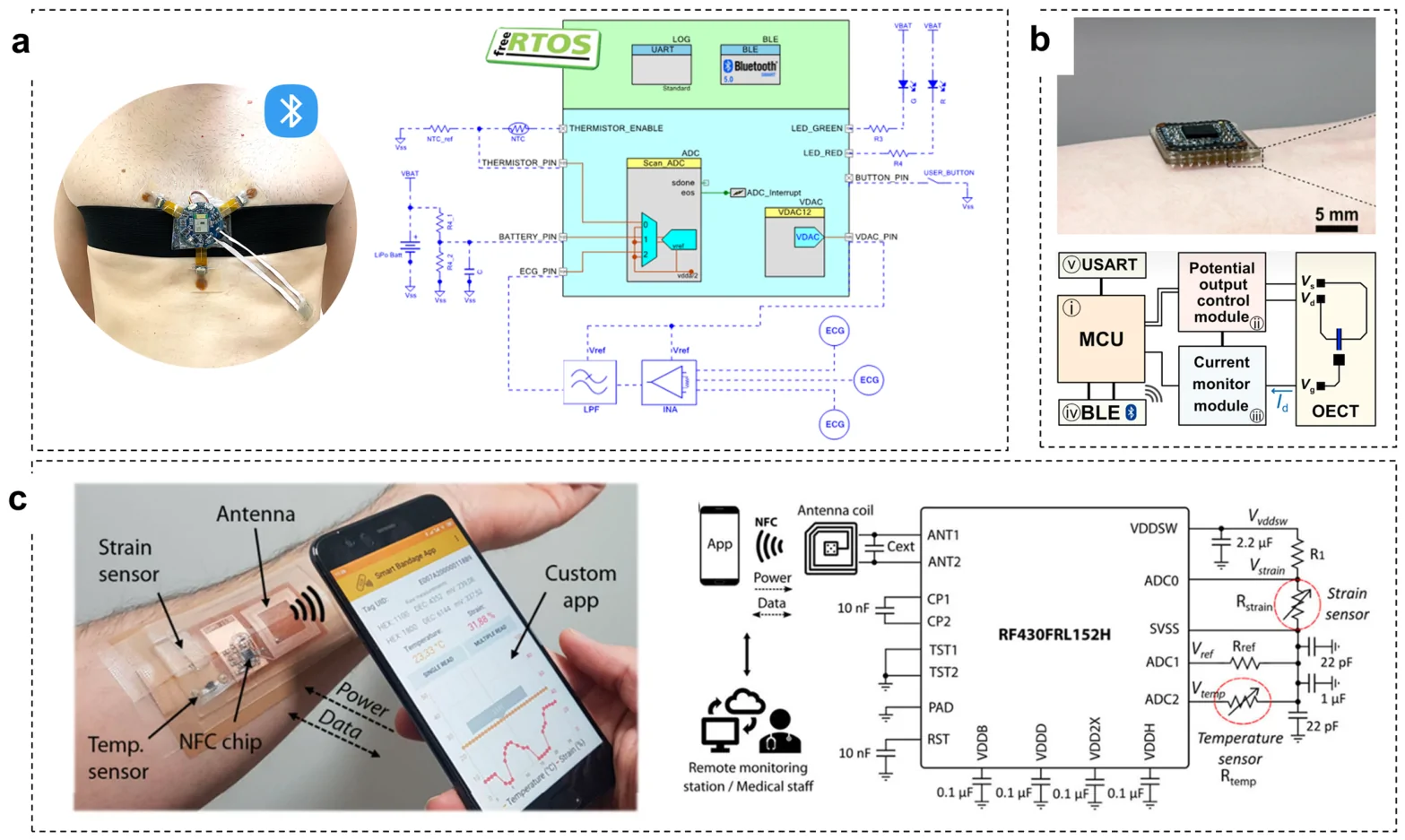
(**a**) BLE wearable for single-lead ECG and body temperature monitoring based on the PSoC63 SoC from Infineon Technologies AG, adapted with permission from the corresponding author from [8]. (**b**) BLE wearable with a microneedle array for subcutaneous glucose monitoring based on the ADuCM355 MCU from Analog Devices, adapted from [40]. (**c**) Battery-less NFC wearable with body-temperature and strain sensor based on the RF430FRL152H sensor transponder from Texas Instruments, adapted from [67].

Moreover, BLE is the current gold standard for those applications that require continuous monitoring due to its low-power consumption, widespread adoption, interoperability, and adequate data rate. There are many well-established SoCs supporting BLE, among which stands out the nRF52 and nRF53 series from Nordic Semiconductors (Trondheim, Norway), as well as the STM32 family from ST microelectronics and the PSoC63 from Infineon Technologies AG. Many of these SoCs are also compatible with many others 2.4 GHz-based protocols, such as Zigbee, Thread or ANT+. Among them, the PSoC63 stands out for its reconfigurable analog domain, which facilitates the design and implementation of many front-ends for analog sensors [8,66]. Examples of BLE-based WHMD are shown in Figure 5a,b, while a battery-less NFC-based WHMD is depicted in Figure 5c.

Moreover, beyond short-range protocols such as BLE or NFC, long-range cellular IoT standards are increasingly relevant for remote health monitoring. Narrowband IoT (NB-IoT) and LTE-M (Long Term Evolution for Machines) are particularly attractive in this context, as they provide low-power wide-area connectivity with extended coverage and reduced energy consumption compared to traditional cellular networks, as in the case of the nRF9160 Cellular IoT System-in-Package (SiP) from Nordic Semiconductors. These protocols enable WHMDs to transmit health data directly to cloud platforms or medical servers without relying on nearby smartphones or hubs, making them suitable for applications such as elderly care, chronic disease management, or continuous patient monitoring in rural areas.

In general, SoC architectures have become central to the design of WHMDs, as they integrate both the MCU and the communication interfaces into a single compact device. This high level of integration reduces size and power consumption while simplifying the design process, which is particularly critical for wearable applications where space and energy are limited. Modern SoCs not only provide the digital and analog peripherals required for sensor interfacing (ADC, DAC, SPI, I^2^C, UART), but also embed wireless communication modules, enabling seamless connectivity without the need for external transceivers. While many sensors can be interfaced directly through these peripherals, others require dedicated front-end circuits for signal conditioning. In some cases, this circuit can be as simple as a voltage divider or a Wheatstone bridge, as occurs for resistive sensors. However, other sensors like capacitive sensors or electrochemical sensors require more complex solutions. As presented in Section 2, many wearable sensors are based on electrochemical cells requiring a potentiostat for their readout. In the context of WHMDs, several embedded solutions exist for this purpose, supporting potentiostatic control, amperometric, voltammetric, and impedance-based measurements. Notable examples include the EmStat Pico module from PalmSens BV (Houten, The Netherlands) developed in collaboration with Analog Devices (Wilmington, MA, USA), as well as the AD5940 and ADuCM355 sensor interfaces from Analog Devices, and the LMP91000 low-power electrochemical cell monitor from Texas Instruments.

Similarly, dedicated AFEs are required for ECG acquisition, since the low-amplitude signal (hundreds of µV to a few mV) is highly susceptible to power-line coupling, motion artifacts, and DC electrode offset. This has driven ECG AFEs toward highly integrated CMOS designs combining low noise, high common-mode rejection (via driven-right-leg circuits and active shielding), and high input impedance for dry-electrode compatibility [68]. Commercially, this is exemplified by Analog Devices’ ADAS1000 family, five-electrode ECG AFEs integrating driven right-leg drive, shield driving, AC/DC lead-off detection, and a digitized SPI output. On the research side, ECG AFEs are also trending toward greater reconfigurability and integration, some exploit the cardiac cycle’s quasi-periodicity to dynamically trade fidelity for power without sacrificing feature extraction [69], while others integrate ECG with complementary modalities like photoplethysmography (PPG) and add fast-recovery circuitry to improve robustness after electrode reconnection [70]. As with the electrochemical readout ICs above, these examples reflect a broader shift toward the AFE as an active, adaptive component rather than a fixed signal-conditioning stage.

### Challenges and Future Directions

Despite the remarkable advances in low-power microelectronics and wireless communication technologies, the processing and communication subsystems remain one of the main bottlenecks limiting the widespread deployment of on-skin wearable health-monitoring devices. Future progress will not only depend on developing more powerful electronics, but also on rethinking how physiological information is acquired, processed, transmitted, and ultimately integrated into sustainable wearable platforms.

One of the central challenges is the increasing volume of data generated by modern wearable sensors. The incorporation of multimodal sensing capabilities frequently requires high sampling rates, continuous signal acquisition, and increasingly sophisticated processing algorithms. While these approaches can improve diagnostic performance, they inevitably increase computational complexity and energy consumption. In our view, future wearable systems should move away from the paradigm of continuously collecting and processing all available information toward more intelligent sensing strategies capable of adapting sampling rates and computational resources to the physiological context and clinical relevance of the measured signals.

Closely related to this aspect is the communication strategy employed by current wearable systems. Continuous wireless transmission is often assumed to be the default solution for remote monitoring; however, maintaining active communication links and transmitting large volumes of raw data can become one of the most energy-intensive operations in the entire device [71]. From a healthcare perspective, the objective is not necessarily to transmit every acquired sample but rather to provide clinically meaningful information. Therefore, the challenge is not simply to move computations from the cloud to the wearable device, but rather to develop lightweight and energy-aware algorithms capable of extracting clinically relevant information using the minimum possible sensing, processing, and communication resources. Then, future WHMDs may increasingly evolve toward event-driven architectures in which communication is triggered only when relevant physiological changes, anomalies, or predefined thresholds are detected. Such approaches could significantly reduce power consumption while maintaining the clinical value of the monitored information. For instance, recent work on wearable cardiovascular patches has quantified this trade-off directly, showing that running a lightweight deep-learning model on-device can be more energy-efficient than continuously streaming raw signals over BLE [72].

Beyond power and computational constraints, the wireless transmission and cloud-based storage of physiological data raise important data security and privacy concerns that should also be addressed in wearable system design. Continuous streams of ECG, sweat biomarker, or other physiological data are attractive targets for interception or unauthorized access, and compromised firmware or communication links can expose sensitive health information or even affect device functionality. Processing physiological data locally, rather than transmitting raw signals, can simultaneously reduce power consumption and limit the exposure of sensitive information. However, on-device processing alone does not eliminate the need for robust authentication, encryption, and secure Over-The-Air (OTA) updates. In our view, future WHMD architectures should treat security and privacy as first-class design constraints rather than as an afterthought addressed only at the software or cloud-infrastructure level. In this regard, hardware security modules (HSMs) are increasingly relevant for WHMDs, as they can offload security-critical operations from the main processor while remaining compatible with strict power budgets. For instance, Infineon’s OPTIGA™ Trust M from Infineon Technologies AG is a certified HSM that provides hardware-based device identity through pre-provisioned X.509 certificates, ECC/RSA key pair generation, and AES-based cryptographic operations over a low-power I^2^C interface, enabling secure cloud authentication without requiring the host microcontroller to manage sensitive key material directly [73]. Security functionality is also increasingly being integrated directly into wireless microcontrollers themselves. For instance, the ESP32-C6 by Espressif Systems is already the first RISC-V-based microcontroller to achieve Platform Security Architecture (PSA) Certified Level 2 compliance, combining a hardware-isolated Trusted Execution Environment (TEE), secure boot, and dedicated cryptographic accelerators for multiple standard operations like AES, SHA, RSA, and ECC.

Another challenge for on-skin WHMDs concerns the physical integration of electronics within wearable systems. While advances in flexible substrates and soft materials have significantly improved device conformability, most wearable electronics still rely on rigid silicon-based ICs. As a result, one of the most fundamental failure points remains the mechanical and material mismatch between soft biological tissues, compliant polymeric substrates, and rigid electronic components. Repeated bending, stretching, twisting, and daily mechanical loading can generate stress concentrations that compromise electrical interconnections, device reliability, long-term operational stability, and the quality of the skin-device interface. To address this challenge, considerable research efforts are being devoted to strain-limiting architectures, stretchable interconnects, and gradient-modulus materials capable of progressively transitioning the mechanical properties from soft biological tissues to polymeric substrates and finally to rigid electronic components. Examples include island-bridge configurations, where rigid electronic elements are localized on mechanically protected “islands” interconnected through stretchable conductors [74], as well as serpentine and fractal interconnect geometries that distribute mechanical stress more uniformly [75]. In parallel, liquid-metal interconnects and stretchable conductive composites have emerged as promising alternatives to conventional metallic traces due to their ability to tolerate large deformations without electrical failure. Similarly, gradient-modulus adhesives and soft encapsulation layers are being investigated to progressively transition mechanical properties between rigid and soft materials, thereby reducing stress concentrations and improving adhesion under physiological strain [76].

Future designs should therefore focus not only on developing flexible sensors but also on improving packaging technologies, interconnect architectures, and heterogeneous integration strategies capable of mitigating the mechanical mismatch between rigid and soft components. At the same time, an emerging research direction aims to move beyond flexible substrates by developing intrinsically flexible electronic components. Recent advances in thin-film electronics, organic and polymer-based semiconductors, flexible transistors, printable electronics, and intrinsically stretchable circuits are opening new possibilities for reducing the dependence on rigid silicon-based components. Although these technologies currently lag behind conventional microelectronics in terms of performance and technology readiness, they represent a promising pathway toward fully conformable wearable systems in which sensing, processing, communication, and power-management functionalities can all accommodate mechanical deformation.

Finally, sustainability also remains an important challenge for next-generation wearable electronics. Significant efforts have been devoted to developing biodegradable substrates, recyclable polymers, and environmentally friendly fabrication processes. However, true sustainability cannot be achieved by focusing exclusively on the substrate. Current wearable devices still incorporate multilayer electronic assemblies containing complex SoCs, batteries, communication modules, and rare-earth or critical raw materials. Embedding non-recyclable or difficult-to-recover electronic components into biodegradable materials does not necessarily reduce the environmental impact of the final device and may even complicate end-of-life processing. In our view, future sustainable wearable systems should adopt a more co-design approach that considers the complete device life cycle, including material sourcing, manufacturing, repairability, reusability, component recovery, and recycling pathways.

Overall, we believe that future processing and communication units will be defined not only by improvements in computational performance but also by their ability to operate intelligently, efficiently, mechanically robustly, securely, and sustainably. Achieving this goal will require a shift from data-centric architectures toward information-centric systems that prioritize clinically relevant outcomes while minimizing energy consumption, mechanical failure risks, security vulnerabilities, and environmental impact.

## 4. Antennas

As introduced in the previous section, WHMDs rely on wireless communication in Body Area Networks (BAN), also referred to as Medical Body Area Networks (MBAN), to transmit physiological data. Therefore, antenna design becomes a critical aspect of WHMD development, often as important as the choice of communication protocol itself. Wearable antennas must be designed to operate efficiently across the frequency bands required by the selected wireless technology, ranging from the low-MHz range used for NFC and RFID, the high-MHz range (700–900 MHz) for emerging LPWAN cellular standards such as LTE-M and NB-IoT, to the 2.4 GHz ISM band for BLE, Wi-Fi, and ANT+, and extending to several gigahertz for Ultra-Wideband (UWB) applications [77]. Since antenna dimensions scale with the wavelength of operation, lower-frequency protocols require larger antennas to achieve efficient radiation and broader coverage, whereas higher-frequency systems enable smaller form factors but typically support shorter-range communication. Table 1 summarizes the most relevant protocols together with their typical antenna types, operating bands, and key design considerations, as well as some representative examples in the WHMD context [78,79,80,81].

For wearable antenna design, both the conductive trace and substrate material properties play a critical role, as even slight deviations can significantly affect antenna performance. Conductive traces must exhibit high conductivity to minimize resistive losses and maximize radiation efficiency, while substrate parameters such as dielectric constant, loss tangent, and thickness directly influence impedance matching and bandwidth. Consequently, alternatives to traditional copper on FR-4 substrates often rely on highly conductive materials such as AgNPs, AgNWs, or copper inks, typically deposited through printing or additive manufacturing techniques onto FR-4 or flexible plastic substrates (e.g., PET or Kapton). Such approaches enable improved mechanical compliance, reproducibility, and more sustainable fabrication pathways for wearable antennas.

Despite the remarkable advances in wearable antenna design, several challenges remain before wireless communication systems can be seamlessly integrated into fully flexible on-skin WHMDs. While significant efforts have focused on improving antenna flexibility, miniaturization, and radiation efficiency, future developments will increasingly require a system-level perspective in which antennas are co-designed with the sensing, processing, communication, and power-management subsystems.

One of the most persistent challenges in wearable antennas is associated with their interaction with the human body. Since biological tissues exhibit relatively high dielectric constants and electromagnetic losses, antenna performance can be significantly affected when operating in close proximity to the skin. Variations in body morphology, placement location, posture, and movement can modify impedance matching conditions and radiation characteristics, leading to detuning effects and communication instability [82]. Consequently, future designs must prioritize robustness against body-induced variations rather than optimizing performance only under ideal laboratory conditions. Moreover, future wearable antennas must be designed not only to maximize communication performance, but also to comply with increasingly stringent specific absorption rate (SAR) requirements and long-term exposure considerations.

This latter connects with the implications of the regulatory and certification aspects associated with the body-antenna interaction. Wearable antennas operating in unlicensed bands must comply with region-specific emission regulations and SAR compliance is typically verified through standardized testing protocols like IEC 62209 and assessed against limits set by bodies established by organizations such as the International Commission on Non-Ionizing Radiation Protection (ICNIRP), the Institute of Electrical and Electronics Engineers (IEEE), and the Federal Communications Commission (FCC) [82]. This creates a coupling between the antenna-design and regulatory-clearance processes which should be treated as an early and continuous design constraint rather than a final verification step performed after the antenna is finalized.

**Table 1:** Summary of most relevant wireless communication protocols used in WHMS including their typical antenna types, operating frequency bands and key design considerations.

Protocol	Antenna Type	Frequency Band	Design Considerations	Examples
NFC	Planar coil Loop antenna	HF (13.56 MHz)	Magnetic coupling, designed for short range (<4 cm), easy integration into flexible substrates.	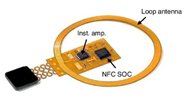 Adapted from [83]
RFID	Planar coil, Dipole	LF (125–134 kHz) HF (13.56 MHz) UHF (860–960 MHz)	HF coils for short-range inductive coupling, UHF dipoles enable longer ranges but difficult miniaturization.	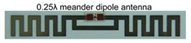 Adapted from [84]
UWB	Planar monopole, Slot antenna	3.1–10.6 GHz	High precision for positioning, they can be miniaturized and are stable under bending, low interference with body tissues.	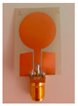 Adapted from [85]
BLE	Planar monopole, Chip, Planar Inverted-F Antenna (PIFA)	2.4 GHz ISM	Compact and lightweight, stable radiation efficiency, often require ground isolation or detuning-mitigation techniques to reduce absorption.	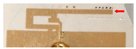 PIFA 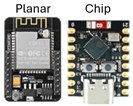 Adapted from [86,87]
WiFi		2.4 GHz ISM 5 GHz		
LTE-M	Monopole, PIFA, patch, multiband antennas	Cellular LTE/GSM	Designed for reliable long-range communication, multiband operation, difficult to tune.	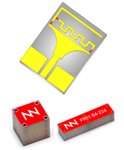 Adapted from [88,89]
NB-IoT				

Mechanical deformation represents an additional challenge. On-skin devices are continuously exposed to bending, stretching, twisting, and compression during daily activities, all of which can alter antenna geometry and electromagnetic behavior. Although flexible conductive materials and stretchable structures have demonstrated promising results, maintaining stable wireless performance under repeated mechanical loading remains an active research area. Future wearable antennas should therefore be evaluated not only from an electromagnetic perspective but also in terms of long-term mechanical reliability and durability.

Another important challenge is the increasing integration of multiple wireless functionalities within the same wearable platform. Future WHMDs are expected to simultaneously support communication protocols such as BLE, NFC, LTE-M, NB-IoT, RFID, and wireless power transfer or RF energy-harvesting systems. While each subsystem may perform adequately in isolation, their coexistence within a compact wearable device can introduce electromagnetic coupling, interference, and mutual detuning effects that degrade overall system performance. In our view, one of the most significant future challenges will not be the design of individual antennas, but rather the reliable integration of multiple wireless technologies within a shared electromagnetic environment. Addressing this issue will require antenna-system co-design strategies that consider communication, sensing, and power transfer requirements simultaneously from the early stages of development. Reconfigurable antennas capable of dynamically adapting their operating frequency, radiation pattern, or impedance characteristics may provide an effective solution for future multi-protocol platforms [90].

A further challenge concerns the coexistence between wireless communication and RF energy harvesting. Since future self-powered wearables may incorporate communication antennas and rectennas within the same device, undesired interactions between power-transfer and communication channels may become increasingly relevant [91]. This situation further reinforces the need for integrated electromagnetic design methodologies that address communication, localization, and energy-harvesting requirements as a unified system rather than as independent components.

From a sustainability perspective, antenna fabrication also presents important opportunities. Current wearable antennas frequently rely on metallic materials and multi-layer manufacturing processes that are difficult to recycle or recover at end-of-life. Future developments should therefore focus on environmentally friendly conductive materials, additive manufacturing approaches, and antenna architectures compatible with circular-electronics principles without compromising electromagnetic performance.

In this context, we believe that the future of wearable antennas will be defined less by the development of individual antenna structures and more by their seamless integration into increasingly complex wireless ecosystems. Achieving reliable operation in future on-skin WHMDs will require co-design approaches that simultaneously address electromagnetic performance, mechanical robustness, multi-protocol coexistence, power efficiency, and sustainability considerations.

## 5. Power Systems

WHMDs usually rely on Li-Po (Lithium Polymer) or Li-ion rechargeable batteries to sustain operation over extended periods of time. These batteries have demonstrated high performance in terms of operating voltage, specific energy, specific power, life cycle and self-discharge rate compared to other battery chemistries. However, they also present some disadvantages that cannot be ignored, such as the toxicity of liquid electrolytes, mechanical rigidity, and the need for frequent charging and periodic replacement [92]. As a result, two major research directions have emerged to address the limitations of current battery-powered WHMDs. On one hand, significant efforts are focused on replacing traditional rigid batteries with flexible and conformable alternatives, emphasizing eco-friendly materials such as solid-state electrolytes, graphene-based structures, and organic polymers to achieve thinner, safer, and more durable energy-storage devices [93]. On the other hand, alternative power-supply strategies based on EH technologies aim to reduce (or even eliminate) dependence on conventional batteries [94]. The long-term vision of both research lines is the development of fully self-powered devices that never require external charging.

**Figure 6:**
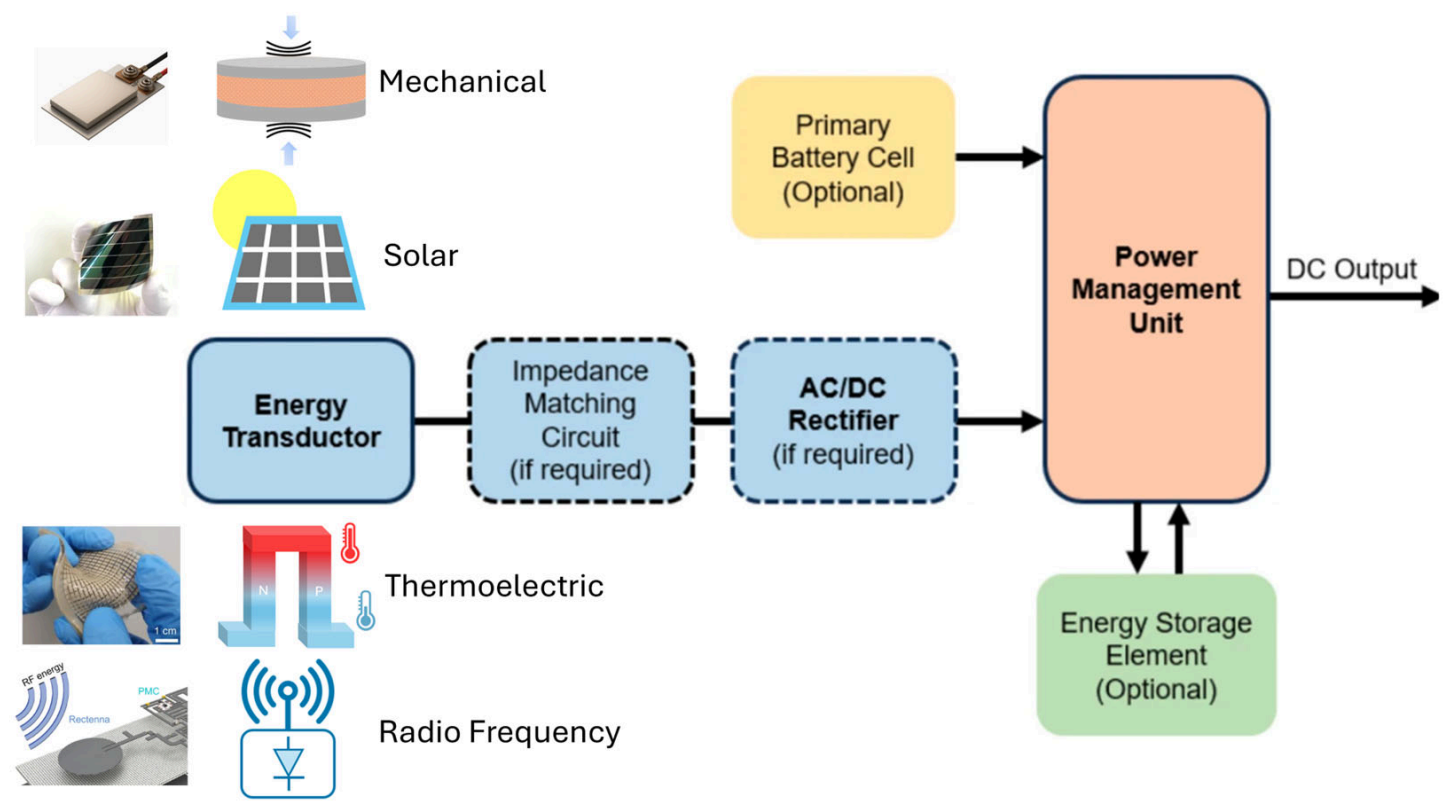
Main components of a single-source energy harvesting system, adapted from [100,101,102,103].

A schematic of a single-source energy-harvesting power supply for a WHMD is shown in Figure 6. The best-case scenario of this approach is when the power consumption of the IoT device is lower than the average harvested power. In that case, the system can operate in harvest-use (HU) mode, enabling battery-less devices capable of continuous operation. This requires a stable and continuous energy source, which is uncommon in most real-world environments. Passive NFC and RFID devices follow this approach by harvesting energy from the electromagnetic field generated by the reader. Because of their limited available power, these systems are typically used in non-critical applications with low sampling rates. For instance, it is used to obtain spot values of body temperature [67], glucose level [95], or gas exposure [96]. In such devices, the coil antenna itself acts as the energy transducer, and only an impedance-matching network (e.g., matching capacitors) is required to set the LC resonance frequency, as most vendors already integrates the PMU in their NFC chips, such as the NGC1081 from Infineon Technologies AG [97], the NTP53x2 from NXP Semiconductors [98], or the RF430FRL152H from Texas Instruments [99].

However, the discontinuous and intermittent nature of most EH sources generally prevent continuous HU operation, making it necessary to incorporate an energy-storage element (e.g., a battery or supercapacitor). This enables alternative operating modes such as harvest-store-use (HSU) or harvest-use-store (HUS) [104]. Moreover, many EHs also consider the use of a primary back-up battery for those critical applications that require continuous operation or that do not allow for long inactive periods. The following sections are then focused on the current trends around energy storage devices and energy harvesting techniques in the context of WHMD.

### 5.1. Energy-Storage Devices

As introduced before, there is an emerging line of research aimed at reducing the environmental impact of batteries and supercapacitors. To achieve this, new electrode materials, substrates, and electrolytes are being explored as sustainable alternatives to current technologies. Despite these developments, Li-ion batteries remain the primary power source for most electronic devices due to their high nominal operating voltage, high energy and power densities, and long cycle life. Consequently, recent research on Li-ion batteries has focused on developing flexible electrodes, electrolytes, and structural designs to meet the mechanical and form-factor requirements of WHMDs [105,106]. However, safety concerns, particularly the risk of flammable liquid-electrolyte leakage, and the challenges associated with miniaturization pose significant limitations for their use in wearable devices.

**Figure 7:**
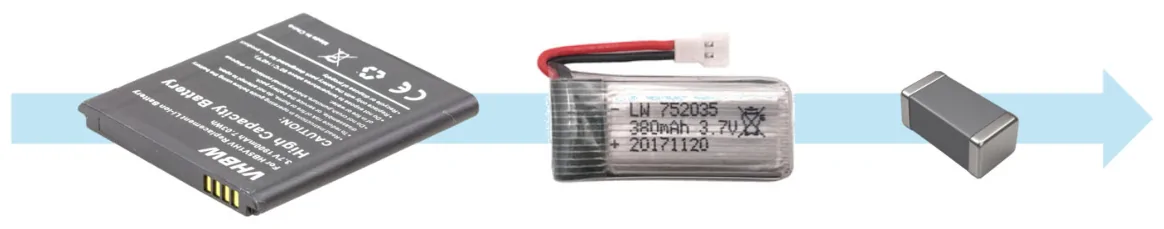
Evolution of battery technology in portable devices, illustrating the transition from Li-ion and Li-Po batteries to next-generation solid-state batteries. Figures extracted from [115,116].

Li-Po batteries emerged as an evolution of Li-ion technology, replacing the liquid electrolyte with a gel-like polymer electrolyte to reduce leakage risks, as illustrated in Figure 7. Although Li-Po batteries exhibit lower energy density, shorter cycle life, and higher self-discharge compared to Li-ion batteries, they enable thinner, lighter, and more conformable designs, making them better suited for WHMD applications. Li-Po batteries are already commercially available in a wide range of sizes, shapes, and thicknesses, and are integrated into many wearable devices on the market. Nevertheless, despite their reduced leakage risk, Li-Po batteries remain flammable, and their soft, lightweight construction makes them more susceptible to swelling, puncture, and mechanical damage. Additionally, disposal regulations for lithium-based batteries in many developed countries are costly and restrictive. Their carbon footprint is also significant, not only due to the energy-intensive manufacturing process but also because lithium batteries are classified as dangerous goods for transportation, requiring special handling and contributing to higher emissions [107,108]. These limitations continue to motivate the search for next-generation energy-storage systems.

In this context, solid-state batteries (SSBs) are gaining increasing attention as one of the most promising alternatives due to their enhanced safety, higher energy density, and longer cycle life [109]. Contrary to the traditional batteries, SSBs use a solid electrolyte for ionic conductions between the electrodes, thus eliminating leaking issues and improving the protection from degradation. In 2020, TDK Electronics (Munich, Germany) successfully commercialized CeraCharge^®^, the world’s first rechargeable all-ceramic solid-state battery. This battery is based on a Li-based ceramic oxide electrolyte/electrode and copper charge collector. With a package of 4.4 mm × 3 mm × 1.1 mm package, similar to Multi-Layer Ceramic Capacitors (MLCC), it is able to offer a 100 µAh nominal capacity at a 1.5 V rating, which is quite promising for WHDM applications [110]. Furthermore, SSBs open the possibility of incorporating biodegradable or otherwise environmentally friendly materials, which is particularly advantageous for end-of-life disposal. As a result, many researchers and manufacturers are focusing on SSB technologies aligned with sustainability initiatives. For instance, zinc/manganese dioxide (Zn/MnO_2_) systems have emerged as preferred eco-friendly alternatives to lithium-based chemistries due to their non-toxicity, abundance, and low environmental impact [111,112,113]. In fact, this is the approach followed by Molex (Lisle, IL, USA) for the development of their commercially available flexible ultra-thin non-rechargeable battery with a nominal voltage of 1.5 V (or 3 V) and a capacity of ~20 mAh [114], being a quite interesting alternative for disposable or short-lived WHMD. Overall, SSBs are expected to play a central role in the next generation of WHMDs due to their sustainability, safety, and performance advantages. As manufacturing processes mature and large-scale production becomes feasible, SSBs are poised to become the natural replacement for current lithium-based batteries.

In parallel to advances in battery chemistry, supercapacitors are increasingly considered as a complementary energy-storage solution for WHMDs. Compared to batteries, supercapacitors offer substantially higher power density, faster charge/discharge rates, and considerably longer cycle life, at the cost of lower energy density. These characteristics make them particularly well suited to buffering the intermittent and variable power delivered by ambient energy harvesters, directly supporting the HSU/HUS operating modes discussed above. Although still far from providing the trade-off between performance and maturity of the previously mentioned devices, a particularly relevant development for on-skin WHMDs is the emergence of hydrogel-based electrolytes, which are enabling supercapacitors that are intrinsically flexible, stretchable, and self-adhesive, properties that allow them to conform closely to the skin rather than relying on rigid packaging [117]. Beyond mechanical compliance, hydrogel electrolytes eliminate the need for the liquid or gel-polymer electrolytes associated with flammability and leakage risks in conventional batteries, offering a safer alternative for direct skin contact. Some hydrogel-based designs further exhibit self-healing capability, allowing the device to recover ionic conductivity and mechanical integrity after minor damage from repeated bending or stretching. Consequently, hybrid architectures that combine a high-energy-density battery with a high-power-density, skin-conformable hydrogel supercapacitor are well positioned to reconcile the long-duration operation requirements of continuous physiological monitoring with the ability to handle transient power demands and mechanical deformation simultaneously.

### 5.2. Energy-Harvesting Systems

The term energy-harvesting refers to the ability to extract energy from the surrounding environment. This technology has been one of the main actors in previous industrial revolutions, thanks to the expansion of green energies, such as solar or wind, which have allowed for escaping from the dependency on fossil fuels. In a similar way, micro- and nano-scale energy-harvesting systems that exploit energy sources naturally available in the operating environment of WHMDs could help reduce reliance on conventional batteries. EH sources can be classified into two main groups depending on whether the energy source for conversion comes from the environmental sources (e.g., solar, thermal, electromagnetic) or from external or user-dependent sources (vibrations, pressure changes, motion, dedicated sources or even physiological processes) [118]. The key element in any EH system is the energy transducer, as it is responsible for collecting the energy from the source and converting it into electrical power. Each energy source presents different power densities which, in turn, depend on multiple factors, such as ambient temperature, weather conditions, location, or even user activity. These dependencies make harvested power unpredictable and discontinuous over time. Moreover, the power density of current EH transducers is relatively low [98], which in most cases restricts WHMDs to HSU operation mode. Additionally, power density is also strongly linked to the physical size of the transducer, posing an additional challenge for WHMDs, where miniaturization is essential.

The complexity of the EH system depends on the selected technology. For instance, while photovoltaic and thermoelectric harvesters already provide a DC voltage at the output, triboelectric, piezoelectric, and RF harvesters generate changing or AC voltages, thus requiring for an AC-DC conversion, i.e., a rectifier. Alternatively, RF harvesters may also require the implementation of impedance matching networks to maximize power transmission to the rectifier and minimize the reflection coefficient [119,120], as shown in Figure 6. Regardless of the harvesting method, WHMDs must incorporate a PMU responsible for regulating, protecting, optimizing the charging and discharging of the energy-storage element, as well as adapting the harvested input power to the output requirements of the application [121]. There are already some semiconductors companies specialized in providing ambient energy PMUs suitable not only for different energy sources, but also different needs in terms of configuration. One of the best positioned companies in this field is E-peas S.A. (Louvain-la-Neuve, Belgium), which counts with an extended range of Ambient Energy Managers (AEMs) optimized for multiple sources, such as photovoltaic, RF, thermal, vibrations and pulsed intermittent sources [122]. While most PMUs share similar functionalities, they differ in the number of input/output channels, configuration flexibility, and support for primary batteries. From a performance standpoint, one of the most critical parameters is the cold-start level, i.e., the minimum input voltage or power required to start the system (typically around 380 mV). Once running, the PMU can continue supplying power to the load as long as the input voltage remains above a certain threshold (typically >50 mV). This requirement introduces one of the main practical challenges in ultra-low-power EH systems. Although an EH may continuously generate power, the harvested voltage is often insufficient to initially activate the PMU, preventing energy extraction and storage. This limitation is particularly relevant for thermoelectric generators operating under low temperature gradients, indoor photovoltaic cells under dim illumination, and low-power RF harvesters, where the available input voltage frequently remains below the PMU cold-start threshold. To overcome this challenge, recent power architectures increasingly incorporate dedicated start-up paths, multi-stage charge pumps, voltage multipliers, or auxiliary energy-storage elements capable of accumulating energy until a sufficient voltage level is reached. Once the system is initialized, the PMU can transition to a more efficient operating mode with significantly lower minimum input-voltage requirements. Consequently, reducing cold-start constraints remains one of the key technological challenges for enabling reliable self-powered WHMDs based on ambient energy harvesting. All this must be align as well with the power-conversion efficiency, which determines how much of the harvested energy is effectively delivered to the device [123,124].

Photovoltaic (PV) cells are among the most attractive energy-harvesting transducers for WHMDs because they offer some of the highest power densities, a critical advantage in size-constrained applications [98]. One of the major benefits of light-based energy harvesting is the maturity of PV technology, which has been widely used for decades. However, its application in WHMDs faces several challenges, including periods of indoor operation under artificial lighting and the need for seamless integration of solar panels into wearable form factors. In addition, PV cells are unsuitable for applications in which the device must remain under clothing, as their operation requires direct light exposure. When it comes to commercial PV cells, monocrystalline silicon (mono-Si) remains the most widely adopted PV technology due to its high efficiency and compact size, making it well suited for miniaturized wearables. Although these solar cells are traditionally rigid, recent fabrication techniques have enabled flexible and semi-flexible mono-Si formats [125]. Leading manufacturers include AnySolar Ltd. (Yongin, Republic of Korea) and PowerFilm, Inc. (Ames, IA, USA). AnySolar’s iXOLAR™ series offers thin SMD-type PV cells capable of generating 23.6 mW (5.9 mA @ 4.46 V) in less than 2 cm^2^ (e.g., KXOB25-02X8F-TB). PowerFilm’s Wireless Electronics series provides PV cells optimized for indoor portable applications, though with lower power density compared to the iXOLAR™ series. In research, Organic Solar Cells (OSCs) are attracting increasing attention due to their lightweight nature, low cost, high tolerance to low-light conditions, and excellent scalability. These features, together with the possibility of being fabricated on flexible substrates, make OSCs a preferable technology for future WHMDs. However, despite rapid progress, flexible OSCs still exhibit lower efficiencies than rigid devices, primarily due to limitations in transparent electrode performance and processing constraints [126,127].

Other promising technologies for WHMDs are based on mechanical EH, either by piezoelectric or triboelectric generation. Although both approaches follow the conversion of biomechanical energy generated by human motion into electrical energy, they rely on different physical principles. While piezoelectric generators are based on materials that generate an electric charge when subjected to mechanical strain or pressure stress due to internal charge displacement within the crystal lattice, the triboelectric generation is produced when two materials with different electron affinity are subject to friction or temporary contact, thus inducing a charge exchange between them [128,129,130]. Unlike PV cells, both technologies produce alternating current and therefore require rectification and conditioning circuits [131,132]. In general, piezoelectric generators count with higher TRL, though recently the focus is shifting to triboelectric due to their higher power output and conversion efficiency [128]. In the context of WHMDs, both technologies are promising for integration into shoe insoles, where they can harvest energy from the mechanical pressure generated during walking. Alternatively, flexible piezoelectric generators are being explored for harvesting energy from joint movements, such as finger, wrist, elbow, or knee bending, although this research is still in early stages [133]. Furthermore, the ability of triboelectric devices to produce energy from friction make them perfect candidates for the fabrication of smart textiles due to the contact and movement that they naturally experience. This is why many leading research groups are developing triboelectric-based textiles using advanced nanomaterials, enabling seamless integration of energy sources into everyday garments such as shirts, jeans, socks, and even bed sheets [134,135].

Thermoelectric generation has also been a widely studied field in last years, especially the Wearable thermoelectric generators (WTEGs) aimed at converting body heat into electricity via the Seebeck effect [136]. Therefore, one of the main challenges of this approach is that the produced energy is completely associated with the temperature gradient between the body (hot side) and the ambient temperature (cold side). As an advantage, WTEGs already produce a DC steady voltage, which facilitates the power conditioning and therefore their integration in final devices. In addition, although they are not still commercially available, multiple studies report the development of flexible WTEGs with good performances and possibilities of integration [137,138].

Moreover, an EH approach which has been attracting increasing attention in the last few years is based on the capability of converting the RF signals into electricity, i.e., the RF harvesting. The massive increase in IoT devices has also come with a large occupation of the electromagnetic spectrum; therefore, there are more and more devices around us that are continuously transmitting information to surrounding devices at different frequencies bands (Wi-Fi, Cellular, Bluetooth, Satellite, etc.). Contrary to the previously mentioned NFC or RFID harvesters, these bands correspond to far-field energy transfer, so the underlying concept around RF harvesting in these bands aims to capture this ambient “ignored” energy to power low-duty-cycle and low-power devices. In this case, the energy transducer is the receiving antenna that is excited by the incoming RF signal producing an AC signal. This signal is then rectified with a diode to obtain the DC output. Additionally, as shown in Figure 6, an RF matching network is required to transform the antenna impedance to the rectifier input impedance at the frequencies of interest, forming a rectenna (rectifying antenna) [139]. A major challenge in RF harvesting for WHMDs is the significant free-space path loss, which depends on transmission frequency, antenna gain, and distance between transmitter and receiver. To maximize the wireless power transfer, it is more interesting to design the RF harvester for lower frequency signals, as they experience lower attenuation in path loss and material absorption. However, low frequency signals mean higher wavelengths, and therefore larger antennas [140]. As discussed in Section 4, compact chip antennas and flexible antennas are well-established technologies with multiple commercial options. Matching networks can be implemented using either lumped elements (inductors and capacitors) or distributed elements (microstrip lines). Lumped-element designs enable compact, miniaturized integration but rely on rigid discrete components, whereas distributed designs allow fully flexible rectenna implementations at the cost of a larger footprint and additional modeling complexity [141], illustrating a broader design trade-off in which body conformability and user comfort are favored over antenna miniaturization and, consequently, harvested power density. In both cases, a key challenge is matching the antenna impedance to the diode input impedance, which varies with both input power and load conditions [140]. This trade-off between power autonomy and user comfort is not unique to RF harvesting since, as discussed above, maximizing available power, whether through larger rectennas, higher-capacity rigid batteries, or increased antenna gain, generally comes at the expense of the flexibility, breathability, and mechanical conformability required for long-term, comfortable wear, reinforcing the need for co-optimized rather than independently maximized power and form-factor design choices. Finally, similar to hybrid EH systems that combine multiple energy sources, there is growing interest in multiband RF energy-harvesting architectures that aggregate power from multiple antennas tuned to different frequencies [96].

Finally, a promising EH modality with particular relevance to on-skin WHMDs is the enzymatic biofuel cell (EBFC), which converts chemical energy directly from metabolites present in sweat (e.g., glucose and lactate) into electrical energy through enzyme-catalyzed redox reactions at the electrode surface [142]. Unlike the other EH technologies discussed above, EBFCs are particularly attractive for on-skin WHMDs because their output power is often proportional to the underlying metabolite concentration, enabling them to function simultaneously as an energy source and a self-powered biosensor, an approach that directly complements the sweat-based electrochemical sensing discussed in Section 2.3. However, EBFCs often lack peak current for wireless transmission so it is an active area of research where EBFCs can be further optimized. In fact, current research has already demonstrated that the integration of EBFCs into self-powered hybrid systems represents a key step toward sustainable and autonomous wearable biosensing [143].

## 6. Conclusions

Throughout this Perspective, we have examined on-skin WHMDs through the lens of their core building blocks: sensors, processing and communication units, antennas, and power systems. Considered individually, many of the technologies discussed have already matured beyond laboratory demonstrations. However, several concerns also emerge, since the central challenge facing next-generation WHMDs is no longer related to only individual component performance, but also to the co-design across all these different domains that have traditionally been optimized independently.

This shift is visible at every level. On-skin sensors are moving from single-purpose transducers toward multimodal, self-calibrating platforms that fuse physiological signals with computational models to compensate for the very sources of variability, motion, sweat, temperature, that limit standalone sensing accuracy. Processing and communication units, in turn, are being asked to do more than balance sampling rate against power consumption since, as physiological data increasingly traverses wireless links and cloud infrastructure, security and privacy are becoming first-class design constraints rather than software-level afterthoughts, placing HSM and certified low-power microcontrollers alongside energy efficiency as core design metrics. Similarly, antennas can no longer be designed as isolated radiating elements as their performance is inseparable from the body they operate on, the SAR and regulatory constraints this creates, and the growing number of coexisting wireless subsystems they must share the electromagnetic environment with. Power systems complete this picture, converging toward hybrid architectures that pair flexible energy storage with complementary harvesting mechanisms. In our view, the most promising route in this field will likely involve hybrid power architectures that combine flexible energy-storage devices, low-power electronics, and multiple complementary harvesting mechanisms to achieve a practical balance between autonomy, wearability and sustainability.

Table 2 synthesizes the current state, key challenges, and emerging solutions across these four building blocks, alongside two considerations that cut across all of them: clinical translation and sustainability. We include these as separate rows precisely because neither can be resolved within a single building block. Clinical translation depends on the joint maturity of sensing accuracy, processing security, antenna compliance, and power reliability, all validated together as an integrated system rather than as independently benchmarked components, and it must additionally satisfy standardized performance benchmarking and regulatory pathways such as FDA clearance or CE marking under the European MDR. Sustainability faces an analogous cross-cutting barrier since biodegradable substrates address only the most visible part of a WHMD’s environmental footprint, while the integrated circuits, batteries, antennas, and communication modules remain difficult to recycle or recover, meaning that material selection, device architecture, and end-of-life strategy must be addressed jointly rather than sequentially.

Beyond the specific solutions listed in Table 2, this cross-cutting framing points to a broader conclusion: the individual innovations in materials, circuits, antennas, and energy systems will not by themselves close the gap between promising demonstrations and ubiquitous clinical deployment. That gap will only close when these domains are designed together, and when technical excellence is pursued alongside economic and social inclusivity, so that the resulting devices are affordable, interoperable, and usable across diverse populations and resource settings, not only in the most technologically advanced healthcare systems.

Looking ahead, we believe the convergence of flexible electronics, secure and energy-aware processing, co-designed antenna systems, sustainable power architectures, and locally executed artificial intelligence will define the next generation of WHMDs, enabling real-time decision-making and early anomaly detection without compromising user privacy or comfort. Ultimately, the future of on-skin WHMDs lies not in isolated technological breakthroughs, but in the disciplined integration of performance, security, clinical rigor, and environmental responsibility into a single coherent design philosophy, one capable of delivering wearable healthcare technologies that are as trustworthy and sustainable as they are capable.

## Figures and Tables

**Table 2:** Major technological challenges and future directions for next-generation on-skin WHMDs.

Core Element	Current State (High-TRL Technologies)	Key Challenges	Emerging Solutions	Long-Term Vision
Sensors	- Ag/AgCl wet electrodes. - Resistive temperature sensors (RTD/NTC). - Commercial invasive sensors (e.g., glucose). - Screen-printed carbon electrodes.	- Long-term skin adhesion and breathability. - Nanomaterial biocompatibility under long-term use. - Sweat matrix variability and biorecognition-layer degradation.	- Conductive and bio-adhesive hydrogels. - Multimodal sensor fusion. - ML-based drift compensation. - Integrated iontophoresis + microfluidic platforms.	Seamlessly skin-integrated, multimodal, self-calibrating sensors with clinically validated long-term biocompatibility.
Processing & Communication	- BLE/NFC/5G SoCs. - Potentiostat ICs. - Dedicated AFEs.	- Data-volume vs. power-budget trade-off. - On-device processing vs. transmission energy balance. - Data security/privacy. - Mechanical mismatch between rigid ICs and soft substrates.	- Event-driven, context-aware architectures. - Lightweight edge-AI models. - Hardware security modules and PSA-certified MCUs. - Island-bridge/serpentine/liquid-metal interconnects.	Intelligent, secure, information-centric (rather than data-centric) systems built on intrinsically flexible electronics.
Antennas	Flexible multi-band antennas	- Body-induced detuning and SAR/regulatory compliance. - Mechanical deformation affecting radiation performance. - Multi-protocol electromagnetic coexistence. - End-of-life recyclability.	- Reconfigurable antennas. - Antenna-system co-design methodologies. - Environmentally friendly conductive materials and additive manufacturing.	Co-designed, regulation-compliant, multi-protocol antenna ecosystems robust to body and mechanical variability.
Power Systems	- Li-Po/Li-ion batteries. - NFC/RFID-based harvest-use PMUs. - Commercial tiny PV cells.	- Battery flammability, disposal, and carbon footprint. - Low and intermittent EH power density. - PMU cold-start limitations. - Rigidity vs. skin conformability.	- Solid-state batteries. - Hydrogel-electrolyte flexible supercapacitors. - Piezoelectric/triboelectric/thermoelectric/RF/enzymatic-biofuel-cell harvesting. - Hybrid battery-supercapacitor-EH architectures.	Fully self-powered, skin-conformable hybrid power systems with minimal battery dependence.
Sustainability (cross-cutting)	- Biodegradable substrates. - Early-stage recyclable conductive inks.	- Non-recyclable ICs, batteries, and antennas embedded within biodegradable substrates. - Lack of global end-of-life strategies.	- Modular/disassemblable architectures. - Circular-electronics design principles. - Component recovery and reuse pathways.	Fully circular, life-cycle-optimized WHMDs with minimal environmental footprint.
Clinical Translation (cross-cutting)	Individual components validation, but few fully integrated systems clinically validated or regulatory-cleared.	- Standardized performance benchmarking. - Long-term validation across diverse populations. - FDA/CE-MDR regulatory pathways.	- Early integration of regulatory requirements into the design cycle. - Closer engineer-clinician-regulator collaboration.	Clinically validated, regulatory-cleared WHMDs deployed at TRL 8–9 in real-world applications.

## Data Availability

No new data were created or analyzed in this study. Data sharing is not applicable to this article.

## References

[1] Mahato K., Saha T., Ding S., Sandhu S.S., Chang A.-Y., Wang J. (2024). Hybrid Multimodal Wearable Sensors for Comprehensive Health Monitoring. Nat. Electron..

[2] Yang S.-M., Lv S., Zhang W., Cui Y. (2022). Microfluidic Point-of-Care (POC) Devices in Early Diagnosis: A Review of Opportunities and Challenges. Sensors.

[3] Wang C., Liu M., Wang Z., Li S., Deng Y., He N. (2021). Point-of-Care Diagnostics for Infectious Diseases: From Methods to Devices. Nano Today.

[4] Xu J., Chen X., Li S., Luo Y., Deng S., Yang B., Lv J., Tian H., Li X., Shao J. (2026). On-Skin Epidermal Electronics for Next-Generation Health Management. Nano-Micro Lett..

[5] Das A., Babu A., Chakraborty S., Van Guyse J.F.R., Hoogenboom R., Maji S. (2024). Poly(*N*-isopropylacrylamide) and Its Copolymers: A Review on Recent Advances in the Areas of Sensing and Biosensing. Adv. Funct. Mater..

[6] Zhao K., Zhao Y., Qian R., Ye C., Song Y. (2023). Recent Advances in Interactive Mechanosensory Electronics with Luminescence/Coloration Outputs for Wearable Applications. ACS Mater. Lett..

[7] Mondal S., Zehra N., Choudhury A., Iyer P.K. (2021). Wearable Sensing Devices for Point of Care Diagnostics. ACS Appl. Bio Mater..

[8] Toral V., Houeix Y., Gerardo D., Blasco-Pascual I., Rivadeneyra A., Romero F.J. (2024). Graphene-Enabled Wearable for Remote ECG and Body Temperature Monitoring. IEEE Flex. Electron..

[9] Seba A., Istrate D., Guettari T., Ugon A., Pinna A., Garda P. (2017). Thermal-Signature-Based Sleep Analysis Sensor. Informatics.

[10] Shan C., Hu J., Zou J., Zhang A. (2022). Wearable Personal Core Body Temperature Measurement Considering Individual Differences and Dynamic Tissue Blood Perfusion. IEEE J. Biomed. Health Inform..

[11] Wang X., Liu Z., Zhang T. (2017). Flexible Sensing Electronics for Wearable/Attachable Health Monitoring. Small.

[12] Ma L.-Y., Soin N. (2022). Recent Progress in Printed Physical Sensing Electronics for Wearable Health-Monitoring Devices: A Review. IEEE Sens. J..

[13] Xu Y., Fei Q., Page M., Zhao G., Ling Y., Stoll S.B., Yan Z. (2021). Paper-Based Wearable Electronics. iScience.

[14] Shaji S., Anna A., Kiran S., Aboobaker A., Varghese A.J., Nancy P., Ravindran L., Thomas S. (2025). Review on Sustainable Flexible Electronics: Exploring the Potential of Chitosan, Cellulose Starch, Silk Fibroin and Gelatin. Discov. Polym..

[15] Tian Y., Wei Y., Wang M., Wang J., Li X., Qin X., Zhang L. (2025). Ultra-Stretchable, Tough, and Self-Healing Polyurethane with Tunable Microphase Separation for Flexible Wearable Electronics. Nano Energy.

[16] Romero F.J., Rivadeneyra A., Toral V., Castillo E., García-Ruiz F., Morales D.P., Rodriguez N. (2018). Design Guidelines of Laser Reduced Graphene Oxide Conformal Thermistor for IoT Applications. Sens. Actuators A Phys..

[17] Houeix Y., Habboush S., Gomez-Gijon S., Rodriguez N., Romero F.J., Rivadeneyra A. (2024). Flexible Thermistors Based on Laser-Induced Graphene from Polyetherimide. Flex. Print. Electron..

[18] Zeng Y., Chen G., Zhao F., Wu C., Xu L., Pan X., Lin F., Li L., He G., Chen Q. (2023). 3D Printing of High-Temperature Thick Film Platinum Resistance Temperature Detector Array. Addit. Manuf..

[19] Tursunniyaz M., Meredith A., Andrews J. (2023). Aerosol Jet Printed Resistive Temperature Sensors with High Sensitivity. Sens. Actuators A Phys..

[20] Wang Y.-F., Sekine T., Takeda Y., Yokosawa K., Matsui H., Kumaki D., Shiba T., Nishikawa T., Tokito S. (2020). Fully Printed PEDOT:PSS-Based Temperature Sensor with High Humidity Stability for Wireless Healthcare Monitoring. Sci. Rep..

[21] Monea B.F., Ionete E.I., Spiridon S.I., Ion-Ebrasu D., Petre E. (2019). Carbon Nanotubes and Carbon Nanotube Structures Used for Temperature Measurement. Sensors.

[22] Nie S., Cai M., Yang H., Shen L., Wang S., Zhu Y., Song J. (2022). Soft, Stretchable Thermal Protective Substrates for Wearable Electronics. npj Flex. Electron..

[23] Salvatore G.A., Sülzle J., Dalla Valle F., Cantarella G., Robotti F., Jokic P., Knobelspies S., Daus A., Büthe L., Petti L. (2017). Biodegradable and Highly Deformable Temperature Sensors for the Internet of Things. Adv. Funct. Mater..

[24] Kuzubasoglu B.A., Sayar E., Cochrane C., Koncar V., Bahadir S.K. (2021). Wearable Temperature Sensor for Human Body Temperature Detection. J. Mater. Sci. Mater. Electron..

[25] Ma T., Li Y., Cheng H., Niu Y., Xiong Z., Li A., Jiang X., Park D., Zhang K., Yi C. (2024). Enhanced Aerosol-Jet Printing Using Annular Acoustic Field for High Resolution and Minimal Overspray. Nat. Commun..

[26] Gugliandolo G., Alimenti A., Latino M., Crupi G., Torokhtii K., Silva E., Donato N. (2023). Inkjet-Printed Interdigitated Capacitors for Sensing Applications: Temperature-Dependent Electrical Characterization at Cryogenic Temperatures down to 20 K. Instruments.

[27] Fu Y., Zhao J., Dong Y., Wang X. (2020). Dry Electrodes for Human Bioelectrical Signal Monitoring. Sensors.

[28] Jung H.-C., Moon J.-H., Baek D.-H., Lee J.-H., Choi Y.-Y., Hong J.-S., Lee S.-H. (2012). CNT/PDMS Composite Flexible Dry Electrodesfor Long-Term ECG Monitoring. IEEE Trans. Biomed. Eng..

[29] Sharma P., Baloda S., Verma D., Janyani V., Sharma R., Gupta N. (2024). Multiwall Carbon Nanotube/Polydimethylsiloxane Composites-Based Dry Electrodes for Bio-Signal Detection. IEEE J. Flex. Electron..

[30] Baloda S., Sriram S.K., Singh S., Gupta N. (2024). Reduced Graphene Oxide-Polydimethylsiloxane Based Flexible Dry Electrodes for Electrophysiological Signal Monitoring. IEEE Trans. Nanotechnol..

[31] Saleh S.M., Jusob S.M., Harun F.K.C., Yuliati L., Wicaksono D.H.B. (2020). Optimization of Reduced GO-Based Cotton Electrodes for Wearable Electrocardiography. IEEE Sens. J..

[32] Romero F.J., Castillo E., Rivadeneyra A., Toral-Lopez A., Becherer M., Ruiz F.G., Rodriguez N., Morales D.P. (2019). Inexpensive and Flexible Nanographene-Based Electrodes for Ubiquitous Electrocardiogram Monitoring. npj Flex. Electron..

[33] Gerardo D., Houeix Y., Romero F.J., Moraila C.L., Blasco-Pascual I., Pérez-Cadenas M., Morales D.P., Rodriguez N., Salinas-Castillo A. (2024). Optimization of Dry Laser-Induced Graphene (LIG) Electrodes for Electrocardiography (ECG) Signals Monitoring. Appl. Phys. A.

[34] Houeix Y., Gerardo D., Romero F.J., Toral V., Hernandez L., Rivadeneyra A., Castillo E., Morales D.P., Rodriguez N. (2024). Dry Laser-Induced Graphene Fractal-like ECG Electrodes. Adv. Electron. Mater..

[35] Joutsen A., Cömert A., Kaappa E., Vanhatalo K., Riistama J., Vehkaoja A., Eskola H. (2024). ECG Signal Quality in Intermittent Long-Term Dry Electrode Recordings with Controlled Motion Artifacts. Sci. Rep..

[36] Tessier A., Zhuo S., Ameri S.K. (2024). Ultrasoft Long-Lasting Reusable Hydrogel-Based Sensor Patch for Biosignal Recording. Biosensors.

[37] Thap T., Yoon K.-H., Lee J. (2016). Graphite Based Electrode for ECG Monitoring: Evaluation under Freshwater and Saltwater Conditions. Sensors.

[38] Toral V., Castillo E., Albretch A., Romero F.J., García A., Rodríguez N., Lugli P., Morales D.P., Rivadeneyra A. (2020). Cost-Effective Printed Electrodes Based on Emerging Materials Applied to Biosignal Acquisition. IEEE Access.

[39] Carneiro M.R., Majidi C., Tavakoli M. (2022). Multi-Electrode Printed Bioelectronic Patches for Long-Term Electrophysiological Monitoring. Adv. Funct. Mater..

[40] Bai J., Liu D., Tian X., Wang Y., Cui B., Yang Y., Dai S., Lin W., Zhu J., Wang J. (2024). Coin-Sized, Fully Integrated, and Minimally Invasive Continuous Glucose Monitoring System Based on Organic Electrochemical Transistors. Sci. Adv..

[41] Zhang E., Cao Z. (2018). Coated Glucose Sensors Dodge Recalibration. Nat. Biomed. Eng..

[42] Bariya M., Nyein H.Y.Y., Javey A. (2018). Wearable Sweat Sensors. Nat. Electron..

[43] Gao F., Liu C., Zhang L., Liu T., Wang Z., Song Z., Cai H., Fang Z., Chen J., Wang J. (2023). Wearable and Flexible Electrochemical Sensors for Sweat Analysis: A Review. Microsyst. Nanoeng..

[44] Pereira J.D., Monge J., Postolache O. (2024). Measurement and Applications: Electrochemical Sensors and Instruments: Main Characteristics and Applications. IEEE Instrum. Meas. Mag..

[45] Parrilla M., Ortiz-Gómez I., Cánovas R., Salinas-Castillo A., Cuartero M., Crespo G.A. (2019). Wearable Potentiometric Ion Patch for On-Body Electrolyte Monitoring in Sweat: Toward a Validation Strategy to Ensure Physiological Relevance. Anal. Chem..

[46] Goh P.S., Ng B.C., Ismail A.F. (2015). Emerging Applications of Functionalized Carbon-Based Nanomaterials. Chemical Functionalization of Carbon Nanomaterials.

[47] Pinheiro T., De Almeida H.V., Nunes D., Martins R., Fortunato E. (2025). Integrated Iontophoresis and Sweat Sensing via Paper-Derived Laser-Induced Graphene Soft Conductors. Mater. Horiz..

[48] Acevedo C.M.D., Gómez J.K.C., Vasquez C.A.C., Ramos J. (2024). Prostate Cancer Detection in Colombian Patients through E-Senses Devices in Exhaled Breath and Urine Samples. Chemosensors.

[49] Dogan M., Nazir S., Jenkins D., Wei Y., Pan G. (2025). Electrochemical Detection of Aβ42 and Aβ40 at Attomolar Scale via Optimised Antibody Loading on Pyr-NHS-Functionalised 3D Graphene Foam Electrodes. Biosensors.

[50] Payne M.E., Zamarayeva A., Pister V.I., Yamamoto N.A.D., Arias A.C. (2019). Printed, Flexible Lactate Sensors: Design Considerations Before Performing On-Body Measurements. Sci. Rep..

[51] Shin S., Liu R., Yang Y., Lasalde-Ramírez J.A., Kim G., Won C., Min J., Wang C., Fan K., Han H. (2025). A Bioinspired Microfluidic Wearable Sensor for Multiday Sweat Sampling, Transport, and Metabolic Analysis. Sci. Adv..

[52] Lin H., Tan J., Zhu J., Lin S., Zhao Y., Yu W., Hojaiji H., Wang B., Yang S., Cheng X. (2020). A Programmable Epidermal Microfluidic Valving System for Wearable Biofluid Management and Contextual Biomarker Analysis. Nat. Commun..

[53] Chen S., Qiao Z., Niu Y., Yeo J.C., Liu Y., Qi J., Fan S., Liu X., Lee J.Y., Lim C.T. (2023). Wearable Flexible Microfluidic Sensing Technologies. Nat. Rev. Bioeng..

[54] Yu Q., Wu J., Xiao W., Zhao C., Zhang W., Chen Y., Liu Y., Liu H., Zhou J., Ding L. (2025). An Integrated, Wearable Paper Microfluidic Array Device for Multicomponent Electrochemical Sweat Sensing. Cell Rep. Phys. Sci..

[55] Han F., Chen S., Wang F., Liu M., Li J., Liu H., Yang Y., Zhang H., Liu D., He R. (2025). High-Conductivity, Self-Healing, and Adhesive Ionic Hydrogels for Health Monitoring and Human-Machine Interactions Under Extreme Cold Conditions. Adv. Sci..

[56] Wang S., Si W., Guo Z. (2025). Superhydrophobic and Super-Stretchable Conductive Composite Hydrogels for Human Motion Monitoring in Complex Condition. J. Colloid Interface Sci..

[57] Song B., Zhang J., Hao S., Shao C., Fu P., Wen J., Yang J., Cong H., Pan C. (2026). Multifunctional Hydrogel Interfaces: Reshaping the Future of Flexible Electronics. Adv. Mater..

[58] Qi R., Zhi X., Xu X., Liu S., Zhang X., Zhang Y., Xue Y., Wang Y., Li S., Jiang T. (2025). Conductive Hydrogels: Synergistic Optimization of Structure-Property-Application in Wearable Sensors. Mater. Today Chem..

[59] Min J., Tu J., Xu C., Lukas H., Shin S., Yang Y., Solomon S.A., Mukasa D., Gao W. (2023). Skin-Interfaced Wearable Sweat Sensors for Precision Medicine. Chem. Rev..

[60] Shahub S., Upasham S., Ganguly A., Prasad S. (2022). Machine Learning Guided Electrochemical Sensor for Passive Sweat Cortisol Detection. Sens. Bio-Sens. Res..

[61] Zhang Z. (2026). Machine-Learning-Enabled Hydrogel Biosensors for Wearable Health Monitoring. Gels.

[62] Jiang Y., Samuel O., Liu X., Wang X., Idowu P., Li P., Chen F., Zhu M., Geng Y., Wu F. (2018). Effective Biopotential Signal Acquisition: Comparison of Different Shielded Drive Technologies. Appl. Sci..

[63] Wang P., Yi T., Hong Z. (2021). High-Input-Impedance Amplifiers Design for Dry-Electrode Biopotential Acquisition: A Review. Proceedings of the 2021 IEEE 14th International Conference on ASIC (ASICON), Kunming, China, 26 October 2021.

[64] Skoric J., D’Mello Y., Plant D.V. (2024). A Wavelet-Based Approach for Motion Artifact Reduction in Ambulatory Seismocardiography. IEEE J. Transl. Eng. Health Med..

[65] Beach C., Li M., Balaban E., Casson A.J. (2021). Motion Artefact Removal in Electroencephalography and Electrocardiography by Using Multichannel Inertial Measurement Units and Adaptive Filtering. Healthc. Tech. Lett..

[66] Toral V., Romero F.J., Castillo E., Morales D.P., Rivadeneyra A., Salinas-Castillo A., Parrilla L., García A. (2022). A Versatile Wearable Based on Reconfigurable Hardware for Biomedical Measurements. Measurement.

[67] Escobedo P., Bhattacharjee M., Nikbakhtnasrabadi F., Dahiya R. (2021). Smart Bandage With Wireless Strain and Temperature Sensors and Batteryless NFC Tag. IEEE Internet Things J..

[68] Wang S., Yang H., Jiao G., Ren M., Li X. (2026). ECG Analog Front End Circuit-A Review. Measurement.

[69] Mondal S., Hsu C.-L., Jafari R., Hall D. (2021). A Dynamically Reconfigurable ECG Analog Front-End With a 2.5× Data-Dependent Power Reduction. IEEE Trans. Biomed. Circuits Syst..

[70] Wang Y., Miao F., An Q., Liu Z., Chen C., Li Y. (2022). Wearable Multimodal Vital Sign Monitoring Sensor With Fully Integrated Analog Front End. IEEE Sens. J..

[71] Boumaiz M., Ghazi M.E., Bouayad A., Balboul Y., El Bekkali M. (2025). Energy-Efficient Strategies in Wireless Body Area Networks: A Comprehensive Survey. IoT.

[72] Ibrahim M.F.R., Alkanat T., Manthey F., Meijer M., Schlaefer A., Stelldinger P. (2026). On-Device Inference versus Wireless Streaming: Energy-Efficient Multi-Modal Deep Learning for Wearable Cardiovascular Patches. arXiv.

[73] Mesa-Simón M., Escobar-Molero A., Parrilla L., Morales D.P., Álvarez-Bermejo J.A., Romero F.J. (2025). Integration of Hardware Security Modules into BLE Beacons: Fundamentals and Use in a Secure and Private Geofencing Application. Internet Things.

[74] Li K., Shuai Y., Cheng X., Luan H., Liu S., Yang C., Xue Z., Huang Y., Zhang Y. (2022). Island Effect in Stretchable Inorganic Electronics. Small.

[75] Fan J.A., Yeo W.-H., Su Y., Hattori Y., Lee W., Jung S.-Y., Zhang Y., Liu Z., Cheng H., Falgout L. (2014). Fractal Design Concepts for Stretchable Electronics. Nat. Commun..

[76] Li Y., Veronica A., Ma J., Nyein H.Y.Y. (2025). Materials, Structure, and Interface of Stretchable Interconnects for Wearable Bioelectronics. Adv. Mater..

[77] Syazreen Ahmad N. (2024). Recent Advances in WSN-Based Indoor Localization: A Systematic Review of Emerging Technologies, Methods, Challenges, and Trends. IEEE Access.

[78] Cao Z., Lu M. (2025). Advancements in Wearable Antenna Design: A Comprehensive Review of Materials, Fabrication Techniques, and Future Trends in Wireless Communication. Micromachines.

[79] Ali U., Ullah S., Kamal B., Matekovits L., Altaf A. (2023). Design, Analysis and Applications of Wearable Antennas: A Review. IEEE Access.

[80] Hussain R., Abou-Khousa M., Khan M.U., Sharawi M.S. (2022). Multi-Standards Slot-Based Antenna Design for NB-IoT and LTE-M Applications. Proceedings of the 2022 16th European Conference on Antennas and Propagation (EuCAP).

[81] Fernández A., Andújar A., Anguera J. (2023). Reconfigurable Multiband Antenna Booster Element under Metallic Environment for IoT Applications. Proceedings of the 2023 17th European Conference on Antennas and Propagation (EuCAP).

[82] Guido K., Kiourti A. (2020). Wireless Wearables and Implants: A Dosimetry Review. Bioelectromagnetics.

[83] Cho S., Han H., Park H., Lee S.-U., Kim J.-H., Jeon S.W., Wang M., Avila R., Xi Z., Ko K. (2023). Wireless, Multimodal Sensors for Continuous Measurement of Pressure, Temperature, and Hydration of Patients in Wheelchair. npj Flex. Electron..

[84] Marindra A.M.J., Pongpaibool P., Wallada W., Siwamogsatham S. (2017). An Optimized Ink-Reducing Hollowed-out Arm Meander Dipole Antenna Structure for Printed RFID Tags. Int. J. Microw. Wirel. Technol..

[85] Särestöniemi M., Sonkki M., Myllymäki S., Pomalaza-Raez C. (2021). Wearable Flexible Antenna for UWB On-Body and Implant Communications. Telecom.

[86] Chhetry A., Kim H., Kim Y.S. (2024). A Wireless Smart Adhesive Integrated with a Thin-Film Stretchable Inverted-F Antenna. Sensors.

[87] Chang Y.-H., Wu F.-C., Lin H.-W. (2025). Design and Implementation of ESP32-Based Edge Computing for Object Detection. Sensors.

[88] Ali E.M., Awan W.A., Abbas A., Abbas S.M., Mohamed H.G. (2025). Compact Frequency-Agile and Mode-Reconfigurable Antenna for C-Band, Sub-6-GHz-5G, and ISM Applications. Micromachines.

[89] Anguera J., Andújar A., Leiva J.L., Massó O., Tonnesen J., Rindalsholt E., Brandsegg R., Gaddi R. (2021). Reconfigurable Multiband Operation for Wireless Devices Embedding Antenna Boosters. Electronics.

[90] Musa U., Shah S.M., Majid H.A., Abidin Z.Z., Yahya M.S., Babani S., Yunusa Z. (2022). Recent Advancement of Wearable Reconfigurable Antenna Technologies: A Review. IEEE Access.

[91] Rao A.S., Aziz A., Aljaloud K., Qureshi M.A., Muhammad A., Rafique A., Hussain R. (2022). Concomitance of Radio Frequency Energy Harvesting and Wearable Devices: A Review of Rectenna Designs. Int. J. RF Mic. Comp.-Aid Eng..

[92] Rong G., Zheng Y., Sawan M. (2021). Energy Solutions for Wearable Sensors: A Review. Sensors.

[93] Kasprzak D., Mayorga-Martinez C.C., Pumera M. (2023). Sustainable and Flexible Energy Storage Devices: A Review. Energy Fuels.

[94] Williams A.J., Torquato M.F., Cameron I.M., Fahmy A.A., Sienz J. (2021). Survey of Energy Harvesting Technologies for Wireless Sensor Networks. IEEE Access.

[95] Banerjee S., Slaughter G. (2022). Flexible Battery-Less Wireless Glucose Monitoring System. Sci. Rep..

[96] Escobedo P., Fernández-Ramos M.D., López-Ruiz N., Moyano-Rodríguez O., Martínez-Olmos A., Pérez De Vargas-Sansalvador I.M., Carvajal M.A., Capitán-Vallvey L.F., Palma A.J. (2022). Smart Facemask for Wireless CO2 Monitoring. Nat. Commun..

[97] Infineon Technologies AG NGC1081 NFC Tag-Side Controller for Smart Sensing Applications. https://www.infineon.com/cms/en/product/power/contactless-power-sensing-ics/nfc-tag-side-controllers/ngc1081/.

[98] NTAG^®^ 5 Link: NFC Forum-Compliant I^2^C Bridge for IoT on Demand. https://www.nxp.com/products/rfid-nfc/nfc-hf/connected-nfc-tags/ntag-5-link-nfc-forum-compliant-ic-bridge-for-iot-on-demand:NTAG5-LINK.

[99] Texas Instruments RF430FRL152H NFC ISO15693 Sensor Transponder With SPI/I2C Interface and 14-Bit Sigma-Delta ADC. https://www.ti.com/product/es-mx/RF430FRL152H.

[100] Badran G., Lazarov V.K. (2025). From Waste to Resource: Exploring the Current Challenges and Future Directions of Photovoltaic Solar Cell Recycling. Solar.

[101] Lee Y.C., Ramiah H., Choo A., Churchill K.K.P., Lai N.S., Lim C.C., Chen Y., Mak P.-I., Martins R.P. (2023). High-Performance Multiband Ambient RF Energy Harvesting Front-End System for Sustainable IoT Applications—A Review. IEEE Access.

[102] Kou Z., Zhang C., Yu B., Chen H., Liu Z., Lu W. (2024). Wearable All-Fabric Hybrid Energy Harvester to Simultaneously Harvest Radiofrequency and Triboelectric Energy. Adv. Sci..

[103] Gómez-Gijón S., Romero F.J., Toral V., Rivadeneyra A. (2025). Energy Harvesting for Sustainable Electronics: Challenges and Opportunities. MRS Commun..

[104] Yuan F., Zhang Q.T., Jin S., Zhu H. (2015). Optimal Harvest-Use-Store Strategy for Energy Harvesting Wireless Systems. IEEE Trans. Wirel. Commun..

[105] Fang Z., Wang J., Wu H., Li Q., Fan S., Wang J. (2020). Progress and Challenges of Flexible Lithium Ion Batteries. J. Power Sources.

[106] Zhao Y., Guo J. (2020). Development of Flexible Li-Ion Batteries for Flexible Electronics. InfoMat.

[107] Costa C.M., Barbosa J.C., Gonçalves R., Castro H., Campo F.J.D., Lanceros-Méndez S. (2021). Recycling and Environmental Issues of Lithium-Ion Batteries: Advances, Challenges and Opportunities. Energy Storage Mater..

[108] International Air Transport Association (IATA) (2025). Battery Guidance Document Transport of Lithium Metal, Lithium Ion and Sodium Ion Batteries 2025.

[109] Lou S., Zhang F., Fu C., Chen M., Ma Y., Yin G., Wang J. (2021). Interface Issues and Challenges in All-Solid-State Batteries: Lithium, Sodium, and Beyond. Adv. Mater..

[110] Chip-Sized Solid-State Battery Ushers in IoT Revolution. https://www.tdk.com/en/featured_stories/entry_024-solod-state-battery-ceracharge.html.

[111] Chen J., Liang J., Zhou Y., Sha Z., Lim S., Huang F., Han Z., Brown S.A., Cao L., Wang D.-W. (2021). A Vertical Graphene Enhanced Zn–MnO2 Flexible Battery towards Wearable Electronic Devices. J. Mater. Chem. A.

[112] Xiao X., Xiao X., Zhou Y., Zhao X., Chen G., Liu Z., Wang Z., Lu C., Hu M., Nashalian A. (2021). An Ultrathin Rechargeable Solid-State Zinc Ion Fiber Battery for Electronic Textiles. Sci. Adv..

[113] Poosapati A., Ambade R.B., Madan D. (2022). Flexible and Safe Additives-Based Zinc-Binder-Free-Hierarchical MnO2-Solid Alkaline Polymer Battery for Potential Wearable Applications. Small.

[114] Thin-Film Batteries|Molex. https://www.molex.com/en-us/products/printed-electronics/thin-film-batteries.

[115] (2016). Raymond VHBW for HB5V1HV Replacement Li-Ion Battery. https://commons.wikimedia.org/wiki/File:VHBW_for_HB5V1HV_Replacement_Li-Ion_Battery-7120.jpg.

[116] de Fazio R., Cafagna D., Marcuccio G., Visconti P. (2020). Limitations and Characterization of Energy Storage Devices for Harvesting Applications. Energies.

[117] Mahdavian F., Allahbakhsh A., Bahramian A.R., Rodrigue D., Tiwari M.K. (2023). Flexible Polymer Hydrogels for Wearable Energy Storage Applications. Adv. Mater. Technol..

[118] Shaikh F.K., Zeadally S. (2016). Energy Harvesting in Wireless Sensor Networks: A Comprehensive Review. Renew. Sustain. Energy Rev..

[119] Wang J., Chen Z., Li Z., Jiang J., Liang J., Zeng X. (2022). Piezoelectric Energy Harvesters: An Overview on Design Strategies and Topologies. IEEE Trans. Circuits Syst. II.

[120] Moreno-Cruz F., Toral-Lopez V., Cuevas M.R., Salmeron J.F., Rivadeneyra A., Morales D.P. (2021). Dual-Band Store-and-Use System for RF Energy Harvesting With Off-the-Shelf DC/DC Converters. IEEE Internet Things J..

[121] Yau C.-W., Kwok T.T.-O., Lei C.-U., Kwok Y.-K., Di Martino B., Li K.-C., Yang L.T., Esposito A. (2018). Energy Harvesting in Internet of Things. Internet of Everything.

[122] E-Peas Semiconductors Website. https://e-peas.com/.

[123] Dostal F. New Advances in Energy Harvesting Power Conversion|Analog Devices. https://www.analog.com/en/resources/analog-dialogue/articles/energy-harvesting-power-conversion.html.

[124] E-Peas Semiconductors AEM30940 Radio Frequency Energy Harvesting Power Management IC. E-Peas. https://e-peas.com/product/aem30940/.

[125] Kannan N., Gupta N. (2024). Flexible Silicon: Status, Opportunities, and Challenges. IEEE J. Flex. Electron..

[126] Wan J., Zhang R., Li Y., Li Y. (2024). Applications of Organic Solar Cells in Wearable Electronics. Wearable Electron..

[127] Zhu J., Xia J., Li Y., Li Y. (2025). Perspective on Flexible Organic Solar Cells for Self-Powered Wearable Applications. ACS Appl. Mater. Interfaces.

[128] Bairagi S., Shahid-ul-Islam, Kumar C., Babu A., Aliyana A.K., Stylios G., Pillai S.C., Mulvihill D.M. (2023). Wearable Nanocomposite Textile-Based Piezoelectric and Triboelectric Nanogenerators: Progress and Perspectives. Nano Energy.

[129] Ali A., Iqbal S., Chen X. (2024). Recent Advances in Piezoelectric Wearable Energy Harvesting Based on Human Motion: Materials, Design, and Applications. Energy Strategy Rev..

[130] Thainiramit P., Yingyong P., Isarakorn D. (2020). Impact-Driven Energy Harvesting: Piezoelectric Versus Triboelectric Energy Harvesters. Sensors.

[131] Verma P., Naval S., Mallick D., Jain A. (2024). Hybrid Piezoelectric-Triboelectric Biomechanical Harvesting System for Wearable Applications. IEEE Trans. Circuits Syst. II Express Briefs.

[132] Barras R., dos Santos A., Calmeiro T., Fortunato E., Martins R., Águas H., Barquinha P., Igreja R., Pereira L. (2021). Porous PDMS Conformable Coating for High Power Output Carbon Fibers/ZnO Nanorod-Based Triboelectric Energy Harvesters. Nano Energy.

[133] Choudhry I., Khalid H.R., Lee H.-K. (2020). Flexible Piezoelectric Transducers for Energy Harvesting and Sensing from Human Kinematics. ACS Appl. Electron. Mater..

[134] Dong K., Peng X., Cheng R., Wang Z.L. (2022). Smart Textile Triboelectric Nanogenerators: Prospective Strategies for Improving Electricity Output Performance. Nanoenergy Adv..

[135] Zhao Z., Hu Y. (2024). Textile Triboelectric Nanogenerator: Future Smart Wearable Energy-Integration Technology. Adv. Mater. Technol..

[136] Nozariasbmarz A., Suarez F., Dycus J.H., Cabral M.J., LeBeau J.M., Öztürk M.C., Vashaee D. (2020). Thermoelectric Generators for Wearable Body Heat Harvesting: Material and Device Concurrent Optimization. Nano Energy.

[137] Yang S., Li Y., Deng L., Tian S., Yao Y., Yang F., Feng C., Dai J., Wang P., Gao M. (2023). Flexible Thermoelectric Generator and Energy Management Electronics Powered by Body Heat. Microsyst. Nanoeng..

[138] Sattar M., Lee Y.J., Kim H., Adams M., Guess M., Kim J., Soltis I., Kang T., Kim H., Lee J. (2024). Flexible Thermoelectric Wearable Architecture for Wireless Continuous Physiological Monitoring. ACS Appl. Mater. Interfaces.

[139] Wagih M., Weddell A.S., Beeby S. (2020). Rectennas for Radio-Frequency Energy Harvesting and Wireless Power Transfer: A Review of Antenna Design [Antenna Applications Corner]. IEEE Antennas Propag. Mag..

[140] Divakaran S.K., Krishna D.D. (2019). Nasimuddin RF Energy Harvesting Systems: An Overview and Design Issues. Int. J. RF Microw. Comput.-Aided Eng..

[141] Malik B.T., Doychinov V., Zaidi S.A.R., Robertson I.D., Somjit N., Richardson R., Chudpooti N. (2019). Flexible Rectennas for Wireless Power Transfer to Wearable Sensors at 24 GHz. Proceedings of the 2019 Research, Invention, and Innovation Congress (RI2C).

[142] Khan R., Uddin M.A., Lee Y.J., Kim T. (2026). Advancements in Enzymatic Biofuel Cells for Wearable Electrochemical Biosensors: Powering Point-of-Care Health Monitoring. Adv. Sens. Res..

[143] Lee J., Han S., Kwon Y. (2024). Self-Charging Hybrid Energy Devices Collaborated with Enzymatic Biofuel Cells and Supercapacitors. Chem. Eng. J..

